# Mitochondrial Transplantation Preserves Regenerative Capacity of Denervated Skeletal Muscle by Disrupting a Ca^2+^ Overload‐Mitophagy‐Ferroptosis Circuit

**DOI:** 10.1002/jcsm.70416

**Published:** 2026-11-05

**Authors:** Haiqi Ding, Jiagu Huang, Jiayao Zhang, Zhizhen He, Jun Luo, Zhao Lin, Linhai Yang, Yibin Su, Mingjuan Zhang, Fuli Wen, Yuan Lin, Cheng Chang, Hanhao Dai, Xuehui Ge, Jie Xu

**Affiliations:** ^1^ Department of Orthopedics Fuzhou University Affiliated Provincial Hospital, Shengli Clinical Medical College of Fujian Medical University Fuzhou Fujian China; ^2^ Department of Orthopedics Ningde Clinical Medical College of Fujian Medical University Ningde Fujian China; ^3^ Fujian Provincial Clinical Medical Research Center for Spinal Nerve and Joint Diseases Fuzhou Fujian China; ^4^ Ningde Municipal Hospital Ningde Fujian China; ^5^ Clinical Medical Research Center of Provincial Hospital Affiliated to Fuzhou University Fuzhou China; ^6^ Institute of New Materials, Guangdong Academy of Sciences, State Key Laboratory of Special Materials Surface Engineering Guangzhou Guangdong China; ^7^ Guangdong‐Hong Kong Joint Laboratory of Modern Surface Engineering Technology, Guangdong Provincial Key Laboratory of Modern Surface Engineering Technology Guangzhou Guangdong China; ^8^ Engineering Research Center of Reactive Distillation, Fujian Province Higher Education Institutes, College of Chemical Engineering Fuzhou University Fuzhou Fujian China

**Keywords:** calcium overload, denervation atrophy, ferroptosis, mitochondrial transplantation therapy, mitophagy, muscle satellite cells

## Abstract

**Background:**

Denervation‐induced muscle atrophy progresses during the interval between nerve injury and reinnervation, and depletion of muscle satellite cells (MuSCs) contributes to impaired functional recovery. Although mitochondrial dysfunction and impaired quality control are implicated in muscle wasting, whether excessive mitochondrial turnover drives MuSC failure after denervation remains unclear. This study aimed to identify the pathogenic mechanisms underlying MuSC depletion and determine whether mitochondrial transplantation therapy (MTT) preserves regenerative capacity.

**Methods:**

A sciatic nerve transection model was established in mice to induce denervation‐associated muscle atrophy. Mitochondria isolated from differentiated MuSCs were transplanted into denervated gastrocnemius muscles beginning 6 h after injury and repeated every 3 days for 6 weeks. RNA sequencing, calcium imaging, mitochondrial ultrastructural analysis, mito‐mKeima‐based mitophagic flux assessment, flow cytometry and molecular analyses were performed to investigate disease mechanisms and therapeutic effects. In vitro calcium overload models combined with Mdivi‐1 treatment and Pink1 knockdown were used to define the regulatory pathway linking calcium stress, mitophagy and ferroptosis.

**Results:**

Denervated muscle exhibited early calcium dyshomeostasis (Fluo‐4 AM fluorescence increased 6.7‐fold vs. Sham, *p* < 0.0001) and mitochondrial dysfunction within 3 days, followed by iron accumulation (nonheme iron increased 1.5‐fold, *p* = 0.0046) and ferroptotic changes by 2 weeks. Mechanistically, sustained calcium overload activated the Ampk‐Tsc2‐mTorc1 pathway in MuSCs, inducing Pink1‐dependent excessive mitophagic flux. Mito‐mKeima flow cytometry confirmed increased mitophagic flux after denervation, with approximately tenfold elevation at Day 3 and persistent activation at 2 weeks. Excessive mitophagy promoted Fth1 degradation (reduced by 47.5%, *p* = 0.0181) and increased Tfr1 expression (1.9‐fold, *p* = 0.0035), thereby enhancing ferroptotic susceptibility. Inhibition of mitochondrial fission or Pink1 knockdown reduced iron accumulation (Fe^2+^ fluorescence reduced by 58.3% and 69.2%, respectively, both *p* < 0.01) and protected MuSCs from ferroptosis. MTT restored mitochondrial homeostasis by reducing calcium overload (73.7% decrease vs. A23187, *p* < 0.0001), increasing ATP production (36.9%, *p* < 0.01), suppressing excessive mitophagic flux, normalizing iron metabolism (39.9% reduction, *p* = 0.0015) and preserving MuSCs (30.0% increase vs. DN_PBS, *p* = 0.0196). Consequently, MTT attenuated denervation‐induced muscle fibre atrophy and improved functional recovery following delayed nerve repair.

**Conclusions:**

This study identifies an early pathogenic cascade linking calcium overload, excessive mitophagic flux and ferroptotic susceptibility as a critical driver of MuSC dysfunction during denervation‐induced muscle wasting. MTT preserves regenerative capacity by interrupting this degenerative circuit and restoring mitochondrial quality control, providing a potential therapeutic strategy for denervation‐associated muscle atrophy and other muscle wasting disorders.

## 1 Introduction

Denervation‐induced skeletal muscle atrophy represents a convergent pathological end point across diverse neurological conditions, including peripheral nerve injury (PNI), stroke and amyotrophic lateral sclerosis [1, 2]. PNI alone accounts for approximately 1 million new cases annually, and a large fraction of these patients experience denervated atrophy [3, 4]. Despite advances in microsurgical nerve repair and postsurgical rehabilitation, a persistent therapeutic gap remains; anatomically successful reinnervation frequently fails to translate into satisfactory functional recovery [5, 6]. This paradox underscores that muscle fate is determined not at the moment of nerve repair but during the prolonged interval between injury and reinnervation.

Axonal regeneration proceeds at approximately 1 mm/day in humans, meaning proximal injuries may require months to reestablish neuromuscular connectivity [6]. However, the temporal tolerance of skeletal muscle to denervation remains poorly defined. Traditional models emphasize progressive proteolysis as the primary driver of atrophy [7, 8, 9], but this framework cannot explain why proteasome inhibition provides only modest functional benefit [10, 11], nor why starvation and immobilization produce vastly slower atrophy kinetics despite similar proteolytic signatures [12, 13]. These inconsistencies implicate a distinct pathogenic process that irreversibly compromises muscle regenerative potential within a discrete postinjury window.

Muscle satellite cells (MuSCs) constitute this regenerative reserve, governing myofibre repair and maintenance [14, 15]. Although MuSCs transiently proliferate following denervation, their pool undergoes progressive, time‐dependent attrition that ultimately precludes functional recovery [16, 17]. Accumulating evidence attributes this depletion to a spectrum of biological events, including Ca^2+^ overload, impaired energy metabolism, oxidative damage and cell death [18, 19, 20]. Nevertheless, the pathogenic mechanisms that erode MuSC viability during the clinically mandated delay before nerve reconstruction remain obscure, representing an urgent unresolved question.

Mitochondria serve dual roles as energy generators and Ca^2+^ buffers, positioning them as potential hubs linking Ca^2+^ dyshomeostasis to cellular fate decisions [21, 22]. When Ca^2+^ overload exceeds mitochondrial buffering capacity, membrane potential collapses, ATP synthesis declines, and mitophagy is triggered [23]. Although transient mitophagy is protective [24], sustained Ca^2+^ imbalance can lead to mitochondrial depletion and disruption of Ca^2+^ ion homeostasis, resulting in self‐amplifying deterioration loop that intensifies the initiating stress. Whether such a circuit operates in denervated MuSCs and whether it culminates in a specific cell death modality remain unknown. Notably, ferritinophagy, the selective autophagic degradation of the iron storage protein ferritin, can liberate labile iron and sensitize cells to ferroptosis [25].

Given the connection between mitophagy and ferritinophagy through shared autophagic machinery, we hypothesized that excessive mitophagy disrupts iron homeostasis through ferritinophagy, which liberates labile iron to drive lipid peroxidation and ferroptotic MuSC death. In this work, we test whether denervation activates a self‐amplifying cascade that irreversibly depletes the MuSC pool and whether mitochondrial transplantation therapy (MTT) can preserve regenerative capacity when delivered within this critical window.

## 2 Materials and Methods

### 2.1 Cell Treatments

Primary MuSCs were isolated from skeletal muscles of 1‐week‐old mice and expanded in vitro. All subsequent experiments were performed using MuSCs between Passages 3 and 5 to ensure consistent cellular characteristics and experimental reproducibility. A Ca^2+^‐overload model was generated by treating MuSCs with 5‐μM A23187 for 6 h (A23187 group) to recapitulate the denervation‐induced Ca^2+^ stress of satellite cells [26]. MuSCs treated with an equivalent volume of DMSO were applied as controls (Ctrl group). After 6 h the medium was switched to DMEM containing 10% FBS and 1% antibiotics for an additional 12 h. To verify that observed effects were Ca^2+^‐specific, cells were cotreated with 20‐μM Bapta‐am (Bapta‐am group) [27], which fully abolished A23187‐induced Ca^2+^ overload.

To investigate the contribution of Ca^2+^‐triggered mitophagic activation to iron metabolism in MuSCs, cells were treated with Mdivi‐1, a pharmacological modulator of mitochondrial dynamics that has been reported to attenuate stress‐induced mitophagy (Mdivi‐1 group). Following 6 h of A23187 exposure, cultures were switched to fresh medium supplemented with 5‐μM Mdivi‐1 for 12 h. An equivalent volume of DMSO was added to the media of both Ctrl and A23187 groups.

For examining how mitochondrial transplantation influences mitophagy and ferroptosis in MuSCs, MuSCs were first exposed to A23187 for 6 h, then supplemented with mitochondria at 1 × 10^8^/mL (Mito group) and further incubated for 12 h.

### 2.2 Preparation and Quality Assessment of Donor Mitochondria

Donor mitochondria were isolated from differentiated MuSCs. Briefly, differentiated MuSCs were incubated with 100‐nM MitoTracker Red CMXRos (Invitrogen, USA) at 37°C for 30 min, washed twice with cold PBS and harvested by centrifugation. Mitochondria were isolated using the Minute Mitochondria Isolation Kit for Mammalian Cells and Tissues (Invent Biotechnologies, Cat. No. MP‐007) according to the manufacturer's instructions. Briefly, cell pellets were resuspended in Buffer A and incubated on ice for 5 min before being passed through the protein extraction filter cartridge by centrifugation (16 000 ×*g*, 30 s). After low‐speed centrifugation (700 ×*g*, 1 min) to remove cellular debris, the supernatant was mixed with Buffer B and centrifuged at 16000 ×*g* for 10 min to pellet mitochondria. The mitochondrial pellet was washed, resuspended in Buffer B, further purified by centrifugation (8000 ×*g*, 5 min), diluted in cold PBS and centrifuged again (16 000 ×*g*, 15 min) to obtain purified mitochondria. Mitochondrial protein concentration was determined using the BCA Protein Assay Kit (Beyotime, China). All isolation procedures were performed at 4°C or on ice to preserve mitochondrial integrity. The mitochondrial membrane potential was evaluated immediately after isolation using the JC‐1 Mitochondrial Membrane Potential Assay Kit (Beyotime, China) according to the manufacturer's instructions. Only freshly isolated mitochondria with preserved membrane potential were used for transplantation. All isolated mitochondrial preparations were transplanted within 2 h after isolation to preserve mitochondrial viability, functional integrity and respiratory capacity.

### 2.3 Mice Treatments

C57BL/6 male mice obtained from the Shanghai SLAC Laboratory Animal Co. Ltd. were raised in an environment that met specific pathogen free (SPF) criteria, with free access to standard chow and tap water. All animal experiments complied with the ARRIVE guidelines and were conducted in accordance with the National Research Council's Guide for the Care and Use of Laboratory Animals.

Following published protocols [28], a sciatic nerve transection model was used to induce denervation‐induced muscle atrophy. Male mice aged 10–12 weeks were anaesthetized with isoflurane, and the right sciatic nerve was transected (DN group); both stumps were sutured to adjacent soft tissue to prevent reapproximation. In the left limb, the nerve was only exposed without injury to serve as control (Sham group).

To evaluate the therapeutic effect of MTT on denervation‐induced muscle atrophy, mice received intramuscular transplantation beginning 6 h after sciatic nerve transection and repeated every 3 days for 6 weeks (14 administrations in total). Each transplantation consisted of 4 × 10^7^ freshly isolated mitochondria suspended in 80‐μL sterile PBS, which were administered through four evenly distributed intramuscular injections (20 μL per site) across the medial and lateral heads of the gastrocnemius muscle to ensure uniform mitochondrial delivery throughout the denervated muscle. Recipient mice were briefly anaesthetized with 2% isoflurane, and mitochondria were delivered by percutaneous intramuscular injection. Control animals (DN_PBS and S_PBS) received an identical volume of mitochondria‐free PBS following the same schedule. After completion of the 6‐week transplantation period, mice in the DN_PBS and MTT groups underwent sciatic neurorrhaphy and were allowed to recover for an additional 6 weeks before behavioural assessments.

Mice were sacrificed by CO_2_ asphyxiation at predefined intervals, and lower limb skeletal muscle was collected for experimental examination. Animal care adhered to the Guide for the Care and Use of Laboratory Animals.

### 2.4 Additional Materials and Methods

Additional details regarding the methods and materials are provided in the Supplementary materials.

### 2.5 Statistical Analysis

Data are presented as mean ± standard error of the mean unless stated otherwise. Data normality (Shapiro–Wilk) and homogeneity of variances (Brown–Forsythe) were checked prior to further testing. Single‐factor comparisons among ≥ 3 groups were evaluated by one‐way ANOVA followed by Tukey's multiple‐comparisons test. Two‐group analyses were carried out with a two‐tailed, unpaired Student's *t*‐test (Welch's correction applied if variances were unequal). All tests were two‐sided, and *p* < 0.05 was considered statistically significant.

## 3 Results

1 Denervation evokes early Ca^2+^ dyshomeostasis and primes ferroptotic vulnerability in skeletal muscle.

To define the initial molecular events within the early denervation window, we performed transcriptomic profiling, Ca^2+^ imaging and iron metabolism assays in a murine sciatic nerve transection model. RNA‐seq analysis of gastrocnemius muscle harvested at Day 3 postinjury, a time point preceding overt structural atrophy, revealed pronounced transcriptional reprogramming: upregulation of atrophy‐related (Cdkn1a, Fbxo32 and Foxo1), ferroptosis‐priming (Tfrc, Gclm and Hmox1) and autophagy‐related (Mapt, Fbxw7 and Ubqln1) genes, concomitant with repression of Ca^2+^ metabolism (Fzd9, Gtf2i and Xcl1), lipid‐metabolic (Acads, Ocrl and Acat) and oxidative‐phosphorylation (Atp5i, Cox4i2 and Sdhb) gene sets (Figure 1A and Figure S1A). KEGG pathway enrichment distinguished two opposing biological programmes: Upregulated genes were enriched in proteasome, FoxO signalling and ferroptosis pathways, whereas downregulated genes were enriched in lipid metabolism, energy metabolism and mitochondrial assembly (Figure 1B). Gene ontology analysis further revealed that upregulated genes participated in cellular aging, autophagy, iron handling and ubiquitin‐mediated proteolysis, whereas downregulated genes were linked to Ca^2+^ homeostasis, lipid metabolism, redox balance and mitochondrial functional pathways (Figure S1B–D). These transcriptional signatures suggested early disruption of Ca^2+^ handling and mitochondrial integrity, with parallel priming of iron‐dependent cell death pathways. To validate these predictions, we examined cytosolic Ca^2+^ levels in isolated single myofibres. Fluo‐4 AM staining revealed markedly elevated cytosolic Ca^2+^ in denervated fibres (DN) compared to controls (Sham) (Figure 1C and Figure S1E). Transmission electron microscopy demonstrated early ultrastructural evidence of mitochondrial stress: clustered phagophores encircling mitochondria, with deranged or vanished cristae, shrunken organelles and elevated electron opacity (Figure 1D and Figure S1F). The concordance of Ca^2+^ dyshomeostasis with mitochondrial injury prompted examination of mitophagy activation. Quantitative RT‐PCR revealed significant upregulation of key mitophagy genes (Bnip3, Atg5, Pink1 and Park2) in denervated muscle (Figure 1E). Notably, this transcriptional activation preceded both overt myofibre atrophy and MuSC depletion, positioning mitophagy as an early, potentially causative event rather than a secondary consequence of degeneration. Concurrent with these mitochondrial quality control responses, we observed evidence of iron metabolic reprogramming. Ferrous iron (Fe^2+^) fluorescent staining demonstrated significantly elevated intracellular iron in denervated myofibres (Figure S1G). Quantitative iron assays confirmed tissue‐specific iron accumulation: Although serum iron levels remained unchanged, nonheme iron content in gastrocnemius muscle was markedly elevated in denervated animals (Figure S1H). Oil Red O staining revealed increased lipid droplet accumulation (Figure 1F, Figure S1I), consistent with altered lipid metabolism. Molecularly, this iron burden was accompanied by downregulation of the ferroptosis suppressors glutathione peroxidase 4 (Gpx4) and solute carrier family 7 member 11 (Slc7a11), alongside upregulation of long‐chain fatty acid CoA ligase 4 (Facl4) and Nuclear Receptor Coactivator 4 (Ncoa4) (Figure S1J). Collectively, these data establish that denervated skeletal muscle manifests a tripartite early pathogenic signature, Ca^2+^ overload, mitochondrial injury with excessive mitophagy priming and ferroptotic vulnerability, within days of nerve injury, preceding structural degeneration.

2 Time‐dependent erosion of MuSC proliferative competence heralds regenerative failure and muscular atrophy.

**FIGURE 1:**
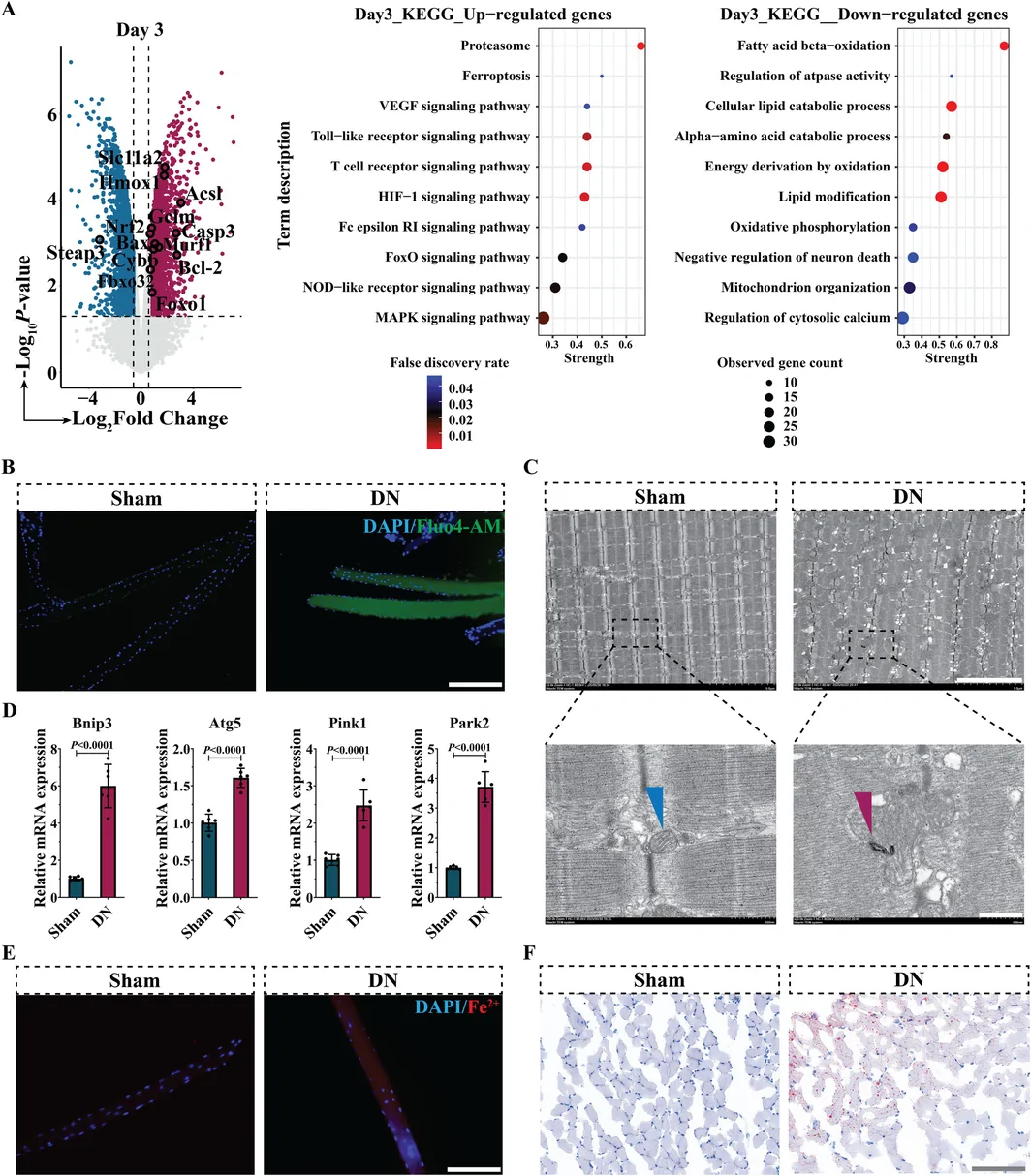
Denervated skeletal muscle exhibits Ca^2+^ dysregulation, enhanced autophagy and ferroptosis at Day 3 postinjury. (A) Volcano plot (left) of differentially expressed genes (DEGs) in gastrocnemius (Gas) muscle at 3 days postdenervation (DN) versus sham‐operated controls (Sham). KEGG pathway enrichment analysis of upregulated (median) and downregulated (right) DEGs. Dot size indicates gene count; colour intensity indicates ‐log10(*p*‐value). (B) Representative Fluo‐4 AM staining (Ca^2+^, green) in isolated Gas myofibres from Sham and DN mice. Scale bar, 40 μm. (C) Representative transmission electron microscopy (TEM) images of Gas muscle from Sham and DN groups of mice. Upper panels: overview (scale bar, 5 μm). Lower panels: mitochondria (scale bar, 500 nm). The blue arrows indicate normal mitochondria, and the red arrows indicate damaged mitochondria. (D) RT‐qPCR analysis of autophagy‐related gene expression (Atg5, Bnip3, Pink1 and Park2) in Gas muscle from Sham versus DN mice, *n* = 4 biological replicates. (E) Representative FerroOrange fluorescence images showing intracellular Fe^2+^ accumulation in single gastrocnemius muscle fibres from DN and Sham mice, together with quantitative analysis of mean Fe^2+^ fluorescence intensity. Scale bar, 40 μm, *n* = 5 mice per group. (F) Representative Oil Red O staining micrographs of Gas muscle from Sham and DN mice, scale bar, 50 μm. Statistical analysis: Two‐sided unpaired *t*‐test. Data are mean ± SEM.

To determine whether the early pathogenic signatures identified above propagate to compromise the muscle stem cell pool, we analysed MuSC dynamics and muscle histology at sequential intervals postdenervation. Transcriptomic profiling of gastrocnemius muscle at 2 weeks postinjury, a stage when early Ca^2+^ and iron disturbances have progressed but structural atrophy remains moderate, revealed marked suppression of MuSC proliferation and differentiation programmes (Myog, Myh10, Sox9 and Cdk2), concomitant with activation of cell death and stress responses (Aimp2, Bcl2, Capn3 and Fas) (Figure 2A). Gene ontology analysis distinguished two opposing trajectories, where the upregulated pathways centred on oxidative stress, cell death and proliferation inhibition, whereas the downregulated pathways governed cell cycle progression, protein metabolism and skeletal muscle regeneration (Figure 2B). This transcriptional shift from anabolic and reparative programmes to catabolic and stress programmes suggested that MuSCs transition from a transient activated state to a functionally compromised, attrition prone phenotype during the second postinjury week. To track this functional transition at single‐cell resolution, we fluorescence‐activated cell sorted (FACS) MuSCs from sham and denervated muscle at Weeks 2 and 4. Quantitative RT‐PCR of proliferation markers, including Cdk4, Ki67, Pcna and Ccna2, revealed a biphasic response. Ki67 and Pcna were upregulated at Week 2, whereas all assessed proliferation genes were significantly downregulated by Week 4 (Figure 2C). This progression from selective to global proliferative shutdown indicates that MuSCs initially attempt reparative expansion but rapidly exhaust their replicative reserve. Flow cytometric quantification confirmed numerical attrition following functional decline. MuSC prevalence (CD45^−^CD11b^−^CD31^−^Sca1^−^Vcam1^+^) showed a significant increase at Week 2 (Figure 2D and Figure S2A,B). By Week 4, MuSC counts fell approximately 15% below Sham values (Figure 2E and Figure S2C), with immunofluorescence confirming markedly fewer Pax7^+^ MuSCs in denervated sections (Figure 2F and Figure S2D). Notably, MuSC numbers continued to decrease at Weeks 6 and 8 after denervation, with a decrease of approximately 38% by Week 8 (Figure S2E), suggesting active cell death or irreversible cell cycle exit rather than sustained differentiation. Additionally, the gastrocnemius wet weight of Sham mice was significantly greater than that of DN mice at this stage (Figure S2F). Neuromuscular junctions exhibited progressive disintegration (Figure S2G), accompanied by fibre type shifting, specifically, loss of fast‐twitch (Type II) myofibres (Figure S2H). Haematoxylin and eosin staining confirmed overt myofibre atrophy at Week 4, characterized by increased fibre density, reduced mean cross‐sectional area, and marked depletion of large‐diameter fibres (Figure S2I). Collectively, the time gap between initial Ca^2+^ disturbances (Day 3) and MuSC proliferative failure (Weeks 2–4) suggests an intervening propagation phase wherein early metabolic stress converts to terminal stem cell attrition. Identifying the molecular executioners of this transition, and whether they operate within a pharmacologically accessible window, is addressed in subsequent experiments.

3 Calcium overload initiates a self‐amplifying mitophagy circuit via Ampk‐Tsc2‐mTorc1 signalling.

**FIGURE 2:**
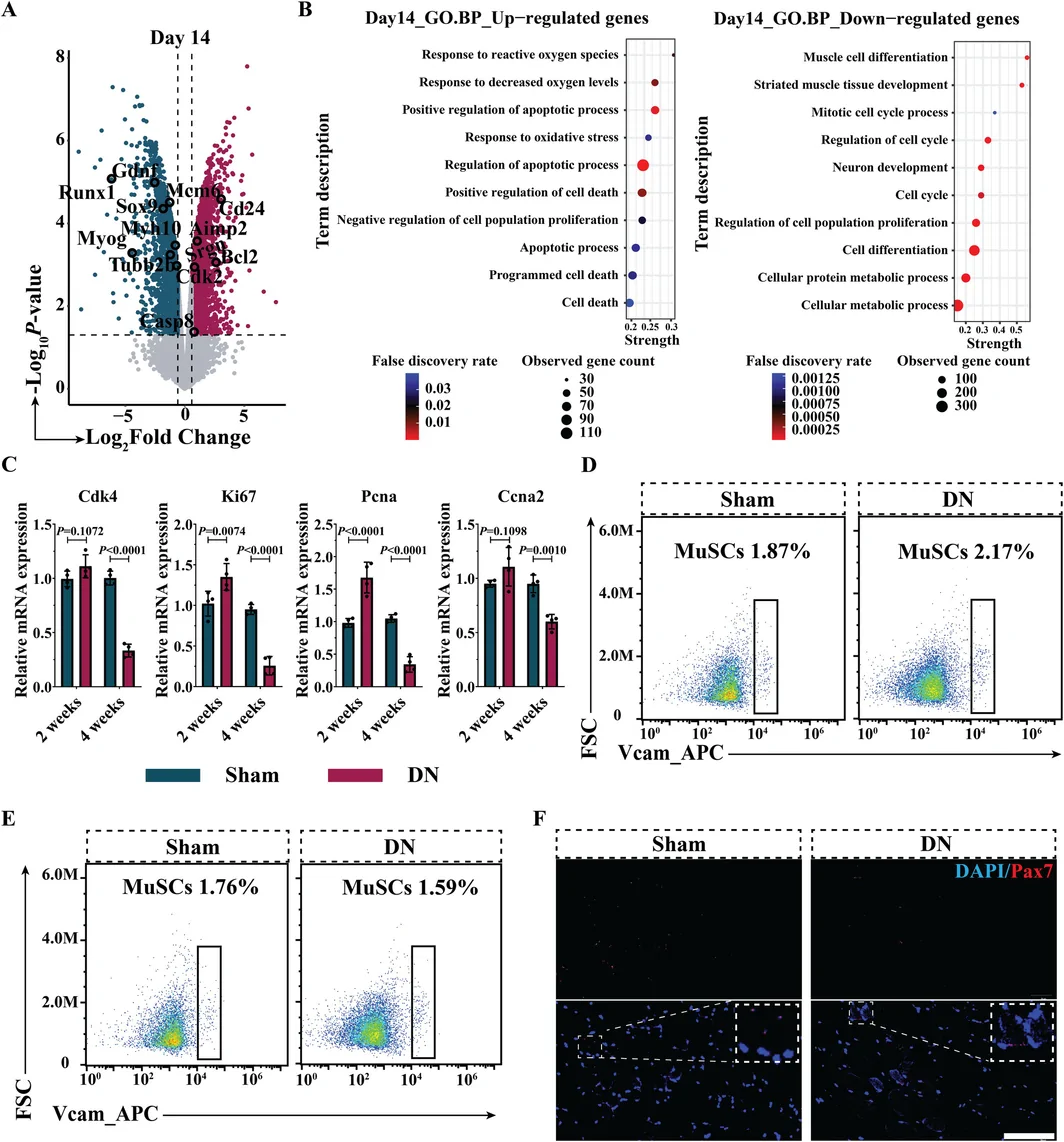
Denervation impairs muscle satellite cell (MuSC) proliferation by 2 weeks. (A) The volcano plot depicts the DEGs in skeletal muscle at 2 weeks postdenervation (DN) versus sham‐operated controls (Sham). (B) GO enrichment analysis of biological process (GO_BP) for upregulated DEGs and downregulated DEGs. Dot size indicates gene count; colour intensity indicates ‐log10(*p*‐value). (C) RT‐qPCR analysis of proliferation‐related gene expression (Cdk4, Ki67, Pcna and Ccna2) in fluorescence‐activated cell sorted (FACS) MuSCs from the hindlimb muscles of Sham versus DN mice at Days 3 and 14, *n* = 4 mice per group. (D,E) Flow cytometric analysis of MuSC abundance (defined as CD45^−^CD11b^−^ CD31^−^ Sca1^−^Vcam1^+^) in gastrocnemius (Gas) muscle from Sham versus DN mice at Weeks 2 (D) and 4 (E). (F) Representative immunofluorescence images of MuSCs in gastrocnemius (Gas) muscle sections from Sham and DN mice, showing Pax7 (red) and DAPI‐stained nuclei (blue). The boxed regions are shown at higher magnification to illustrate Pax7/DAPI colocalization. Scale bar, 100 μm. Statistical analysis: (C) Two‐way analysis of variance test, ANOVA. Data are mean ± SEM.

To identify the molecular executioners propagating damage from early Ca^2+^ dyshomeostasis to time‐dependent MuSC attrition, we investigated how sustained Ca^2+^ overload converts metabolic stress into excessive organelle clearance. We focused on the interval between Day 3 (Ca^2+^ disturbance onset) and Week 2 (proliferative failure), a propagation window wherein intervention might interrupt progressive stem cell deterioration. Denervated muscle exhibited profound bioenergetic compromise. ATP content in gastrocnemius was markedly reduced by Day 3 posttransection (Figure 3A). Concomitantly, immunoblotting revealed activation of the energy‐sensing Ampk axis: elevated phosphorylation of Ampk and its downstream target Tsc2, with reciprocal suppression of phosphorylated mTorc1 (Figure 3B and Figure S3A). This signalling configuration, characteristic of cellular energy crisis, positions Ampk as the molecular link between Ca^2+^‐induced mitochondrial dysfunction and catabolic programme activation. The downstream consequence of mTorc1 suppression was markedly intensified mitophagy. Western blot analysis demonstrated significant upregulation of Pink1, Parkin and Lc3b‐II in denervated muscle (Figure 3C). Notably, this mitophagy activation occurred while myofibres remained structurally intact, suggesting that organelle clearance is an early, active response to Ca^2+^‐driven bioenergetic stress rather than a terminal degradation process. To establish causality between Ca^2+^ overload and mitophagy activation and to test whether this cascade operates cell‐autonomously in MuSCs, we developed an in vitro model. Primary MuSCs were treated with A23187, a Ca^2+^ ionophore that recapitulates denervation‐induced cytosolic Ca^2+^ stress. Fluo‐4 AM staining confirmed robust Ca^2+^ elevation, which was fully abolished by cotreatment with Bapta‐am, a selective Ca^2+^ chelator (Figure 3D and Figure S3B). A23187 treatment precisely phenocopied the denervation‐induced bioenergetic and signalling signature. JC‐1 staining revealed dissipation of mitochondrial membrane potential (ΔΨm) (Figure 3E, Figure S3C), with concomitant ATP depletion (Figure S3D). Immunoblotting demonstrated identical Ampk‐Tsc2‐mTorc1 activation: elevated p‐Ampk and p‐Tsc2, suppressed p‐mTorc1 (Figure 3F). Quantitative RT‐PCR confirmed upregulation of mitophagy transcripts (Bnip3, Atg5, Pink1 and Park2) (Figure S3E). Crucially, Ca^2+^ chelation with Bapta‐am fully quenched this entire signalling cascade; restoring ΔΨm, ATP levels and mTorc1 activity; and suppressing mitophagy gene expression. These findings establish Ca^2+^ overload as an obligatory trigger of the bioenergetic‐autophagy axis. The ensuing mitochondrial dysfunction, acting as a signal of perturbed energy metabolism, drives sustained mTorc1 inhibition and progressive mitophagy. In turn, mitophagy further compromises both ATP synthesis and Ca^2+^ buffering capacity, thereby amplifying the Ca^2+^‐initiated cascade. Nevertheless, whether this self‐amplifying loop resolves through reversible metabolic adaptation or culminates in terminal cell death, and whether it represents a critical interventional node for preserving stem cell viability during the early denervation window, remains to be determined.

4 Excessive mitophagy executes MuSC depletion via ferritinophagy and ferroptosis.

**FIGURE 3:**
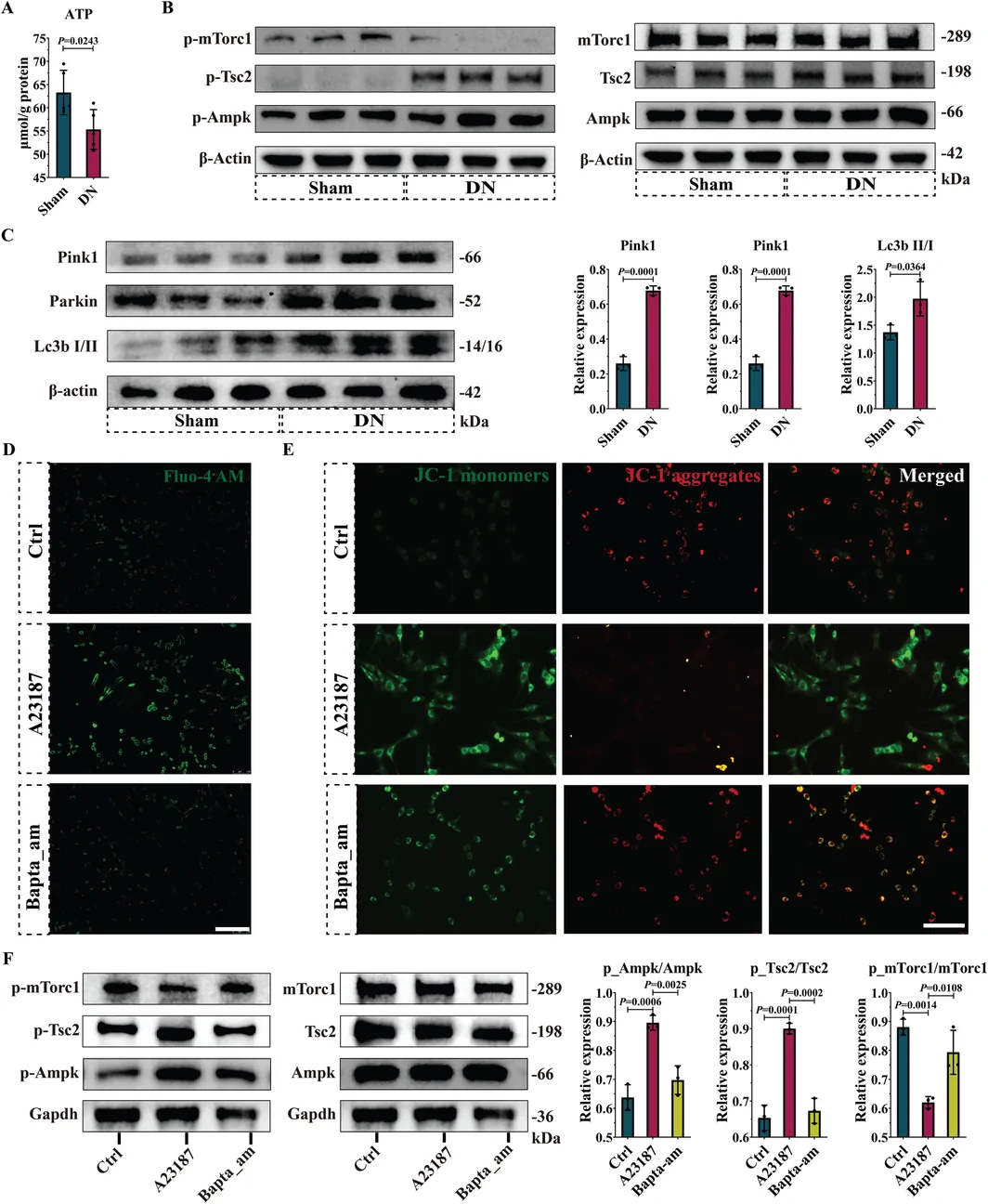
Ca^2+^ overload induces Mitophagy in MuSCs via the Ampk‐Tsc2‐mTorc1 axis. (A) Statistical analysis of the difference in ATP production in the gastrocnemius (Gas) between Sham and DN mice, *n* = 5 mice per group. (B) Representative immunoblots and quantitative analyses of phosphorylated Ampk (p‐Ampk), total Ampk (Ampk), phosphorylated Tsc2 (p‐Tsc2), total Tsc2 (Tsc2), phosphorylated mTorc1 (p‐mTorc1) and total mTorc1 (mTorc1) in Gas from Sham and DN mice. (C) Representative immunoblots and quantitative analyses of mitophagy markers (Pink1, Parkin and Lc3bII) in Gas from Sham and DN mice, *n* = 3 biological replicates. (D) Representative Fluo‐4 AM staining of primary MuSCs treated with vehicle (Ctrl group), 5‐μM A23187 (A23187 group), or A23187 plus 10‐μM Bapta‐am (Bapta‐am group). Scale bar, 200 μm. (E) Representative JC‐1 staining showing mitochondrial membrane potential (ΔΨm) of Ctrl, A23187 and Bapta_am MuSCs in vitro, Red, JC‐1 aggregates (high ΔΨm); green, JC‐1 monomers (low ΔΨm). Scale bar, 50 μm. (F) Representative immunoblots and quantitative analyses of p‐Ampk, Ampk, p‐Tsc2, Tsc2, p‐mTorc1 and mTorc1 of MuSCs in Ctrl, A23187 and Bapta_am groups, *n* = 3. Statistical analysis: (A,C,E) two‐sided unpaired *t*‐test; (F) One‐way analysis of variance test, ANOVA. Data are mean ± SEM.

Given that denervated muscle exhibited profound alterations in iron handling proteins, we examined whether excessive mitophagy disrupts iron homeostasis through autophagic degradation of ferritin, which generates the labile iron pool required for lipid peroxidation and ferroptotic cell death. Western blot analysis revealed marked suppression of ferritin heavy chain (Fth1), the primary iron sequestration protein, concomitant with upregulation of transferrin receptor (Tfr1), the major iron import channel (Figure 4A and Figure S4A). This reciprocal change in Fth1 and Tfr1 expression creates a pathological state in which reduced sequestration capacity combined with enhanced import drives cytosolic labile iron accumulation. Ferrous iron staining demonstrated robust Fe^2+^ accumulation in denervated myofibres (Figure S4B), and flow cytometry confirmed expansion of ROS positive MuSC populations (Figure 4B). The molecular signature of ferroptosis was correspondingly activated, with suppression of the lipid peroxidase Gpx4 and the cystine transporter Slc7a11, alongside induction of Ncoa4 which drives ferritinophagy (Figure 4C). These coordinated alterations in iron loading, antioxidant depletion and pro‐oxidant enzyme induction indicate that ferroptosis serves as the execution modality linking mitophagy to MuSC attrition. To determine whether ferroptotic activation is causally downstream of excessive mitophagy, we employed both pharmacologic and genetic approaches to interrupt mitophagy. In A23187 treated MuSCs, Mdivi‐1 rescued the iron handling phenotype, with restoration of Fth1 levels, attenuation of Tfr1 induction (Figure 4D). Concomitantly, intracellular Fe^2+^ accumulation and ROS generation were suppressed (Figure 4E and Figure S4C,D), lipid peroxidation was prevented as shown by C11 BODIPY red dominance (Figure S4F and Figure S4E), and the ferroptosis molecular signature was normalized with restoration of Gpx4 and Slc7a11 and suppression of Ncoa4 (Figure S4F). To establish mitophagy as the obligate intermediary rather than one of multiple parallel pathways, we employed siRNA‐mediated Pink1 knockdown (siPink1) to suppress Pink1‐dependent mitophagy while preserving Ca^2+^ signalling integrity (Figure S4G). In siPink1 MuSCs, A23187 treatment failed to trigger Fth1 degradation or Tfr1 upregulation (Figure S4H), and intracellular Fe^2+^ remained at baseline levels (Figure S4I). The ferroptosis transcriptional programme was unresponsive, with Gpx4, Slc7a11 and Ncoa4 levels remaining indistinguishable from those in controls (Scr_Ctrl). These findings indicate that excessive mitophagy promotes ferroptosis susceptibility in MuSCs by promoting Fth1 degradation and upregulating Tfr1 expression, thereby leading to the accumulation of labile iron. Collectively, these results establish that the Ca^2+^ overload‐mitophagy cascade operates within the early denervation window to drive MuSC depletion, positioning this axis as a central node for therapeutic intervention against denervation‐induced muscle atrophy.

5 Mitochondrial transplantation disrupts the Ca^2+^‐mitophagy‐ferroptosis cascade in MuSCs.

**FIGURE 4:**
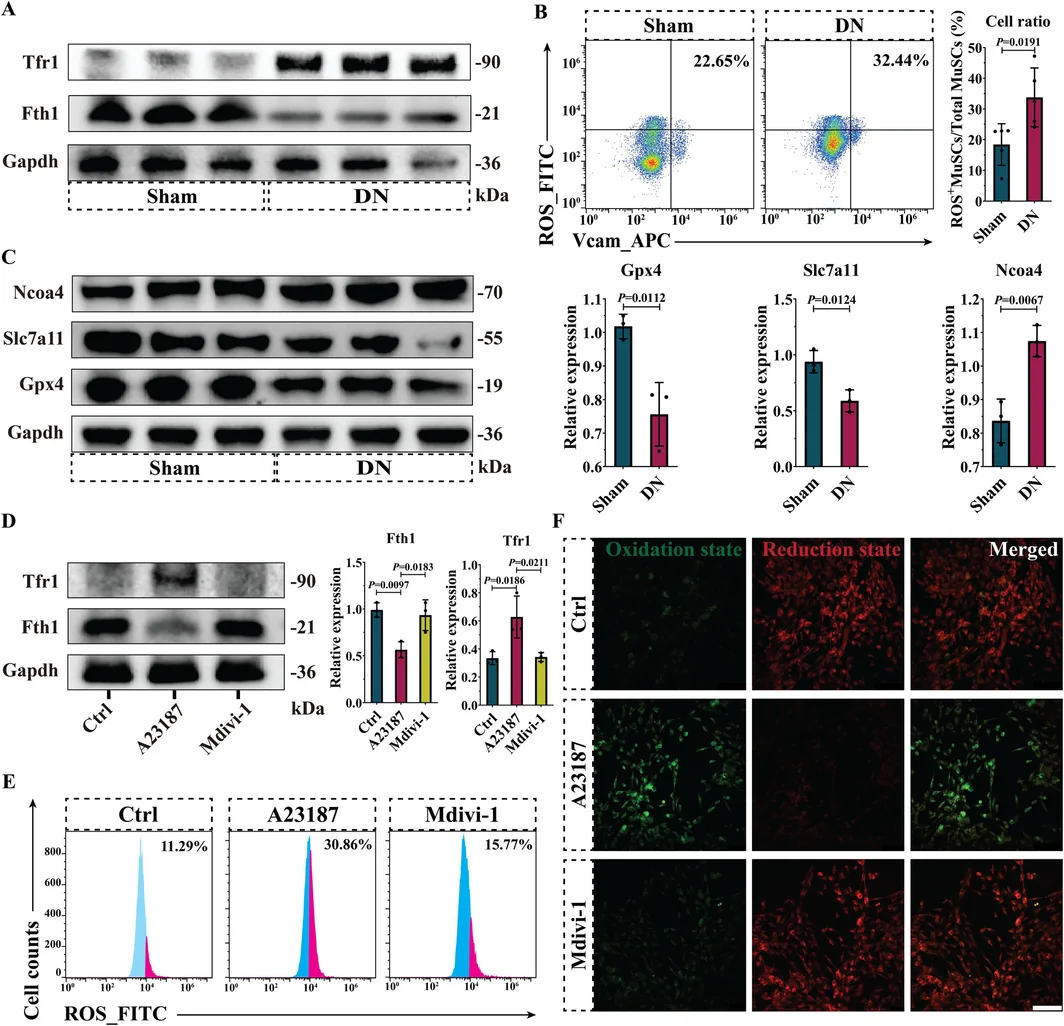
Excessive mitophagy aggravates Fe^2+^ accumulation and triggers ferroptosis. (A) Representative immunoblots of Fe^2+^‐metabolism‐related proteins (Fth1 and Tfr1) in gastrocnemius (Gas) muscle tissue between Sham and DN groups. (B) Representative flow cytometry scatter plots and statistical analysis of ROS^+^ MuSCs in Gas from Sham and DN groups at Day 3, *n* = 4. (C) Representative immunoblots and quantitative analyses of ferroptosis‐related proteins (Gpx4, Slc7a11 and Ncoa4) in Gas muscle between Sham and DN groups, *n* = 3. (D) Representative immunoblots and quantitative analyses of Fe^2+^‐metabolism‐related proteins (Fth1 and Tfr1) in MuSCs of Ctrl, A23187 and Mdivi‐1 groups, *n* = 3. (E) Representative flow cytometry histograms of ROS^+^ MuSCs among Ctrl, A23187 and Mdivi‐1 groups. (F) Representative C11‐BODIPY 581/591 fluorescence images showing the oxidation state of the lipid peroxidation probe in MuSCs from Ctrl, A23187 and Mdivi‐1 groups. Green fluorescence represents the oxidized form of the probe, whereas red fluorescence represents the reduced form. Scale bar, 50 μm. Statistical analysis: (B,C) Two‐sided unpaired *t*‐test; (D) One‐way analysis of variance test, ANOVA. Data are mean ± SEM.

Mitochondria are central to maintaining intracellular energetic and redox homeostasis, while also functioning as critical Ca^2+^ buffers. We therefore hypothesized that transplantation of functional mitochondria could ameliorate Ca^2+^ overload, boost ATP synthesis and potentially attenuate muscle atrophy by suppressing mitophagy driven ferroptosis in MuSCs. Given the enhanced mitochondrial bioenergetics in differentiated MuSCs, primary MuSCs were first induced to undergo differentiation, and mitochondria were subsequently isolated from these cells for MTT. To validate the internalization of donor mitochondria by recipient cells in vivo/in vitro (Figure S5A), the isolated mitochondria were fluorescently labelled with MitoTracker Deep Red FM prior to transplantation (Figure S5B). First, MuSCs were incubated with freshly isolated mitochondria for 24 h, after which MitoTracker Deep Red labelled mitochondria were clearly visualized inside the cells, confirming cellular uptake (Figure S5C). Concurrently, multipoint intramuscular delivery of labelled mitochondria into denervated gastrocnemius resulted in their detection beneath the basal lamina and within sarcoplasm in both transverse and sagittal sections 24 h post injection, verifying myofibre uptake (Figure S5D). These findings establish that exogenous mitochondria are successfully assimilated by skeletal muscle after focal injection. To further examine the capacity of MTT to rescue Ca^2+^ overload, MuSCs were subjected to A23187 treatment, with (Mito group) or without (A23187 group) subsequent supplementation of healthy isolated mitochondria. Twenty‐four hours later, staining with a Ca^2+^ sensitive fluorescent probe revealed a significantly lower mean fluorescence intensity in the Mito group compared to the A23187 group (Figure 5A and Figure S6A). JC‐1 immunofluorescence staining indicated a significantly higher mitochondrial membrane potential in the Mito group relative to the A23187 group (Figure 5B and Figure S6B). Moreover, with the recovery of ATP production (Figure S6C), western blot analysis showed that the phosphorylation levels of Ampk and its downstream effector Tsc2 were significantly reduced, whereas the level of phosphorylated mTorc1 was markedly elevated in the Mito group (Figure 5C). Concurrently, RT‐qPCR revealed a marked reduction in mitophagy associated transcripts within Mito MuSCs (Figure 5D). Fluorescence assays further indicated that Mito MuSCs contained markedly less Fe^2+^ (Figure 5E and Figure S6D) and generated fewer ROS than the A23187 group (Figure S6E). Subsequent FACS analysis corroborated these findings, revealing a pronounced decrease in the percentage of ROS positive MuSCs within the Mito group (Figure S6F). Aligning with the diminished Fe^2+^ and ROS signatures, C11 BODIPY assays revealed a dominant reduced red signal in Mito MuSCs, reflecting substantially attenuated lipid peroxidation relative to A23187 cells (Figure S6G). Western blotting further demonstrated that, relative to the A23187 group, the protein level of Ncoa4 was significantly reduced, whereas the levels of Slc7a11 and Gpx4 were markedly elevated in the Mito group (Figure 5F). Collectively, these data indicate that MTT in vitro can attenuate Ca^2+^ overload triggered mitophagy, preserve mitochondrial bioenergetic and redox homeostasis and consequently diminish the cellular propensity for ferroptosis.

6 MTT restores mitochondrial homeostasis by suppressing denervation‐induced Ca^2+^ overload and excessive mitophagy.

**FIGURE 5:**
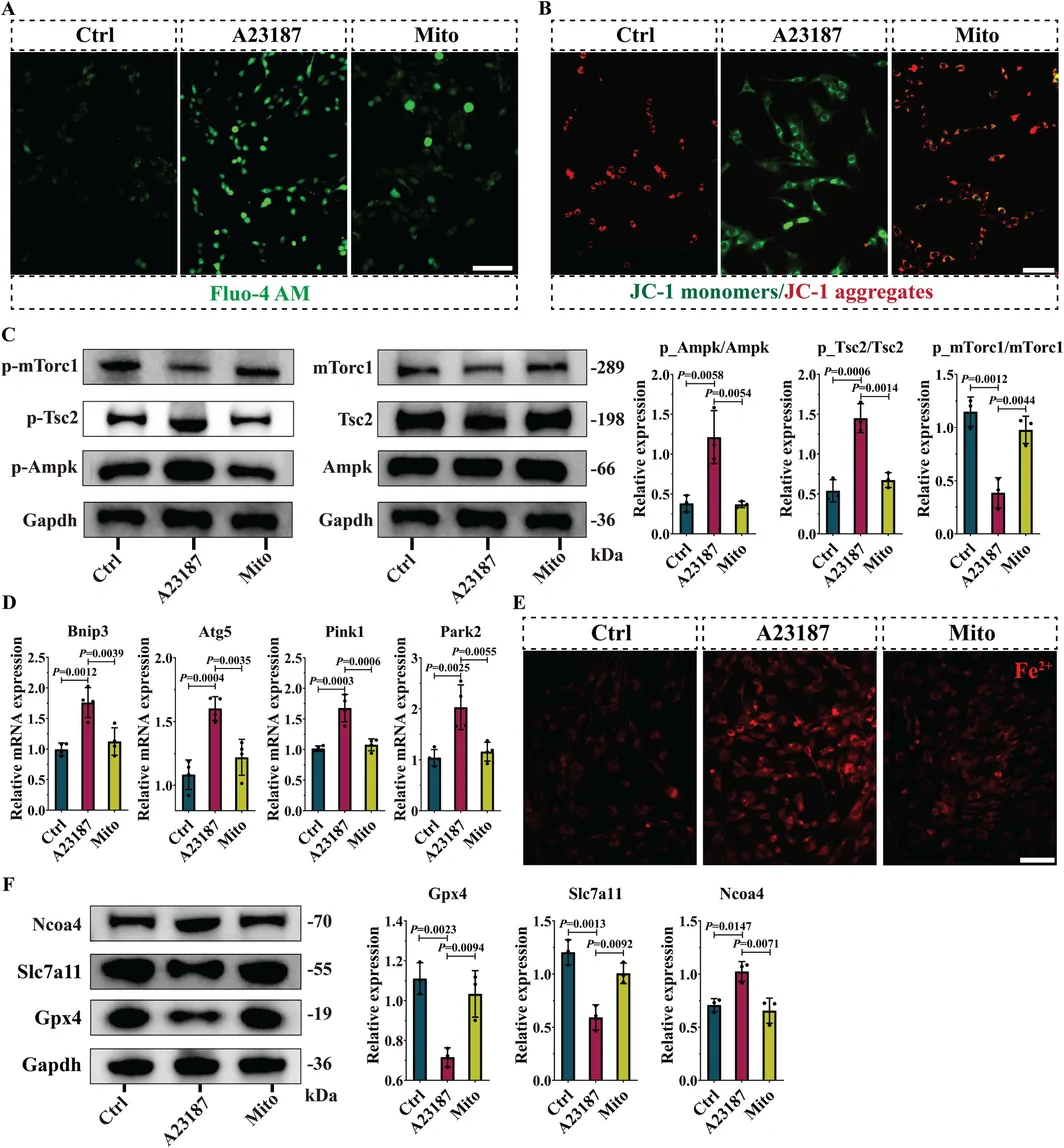
Mitochondrial transplantation therapy (MTT) mitigates Ca^2+^ overload‐induced mitophagy to suppress ferroptosis in MuSCs. (A) Representative Fluo‐4 AM (Ca^2+^, green) staining of MuSCs treated with vehicle (Ctrl group), 5‐μM A23187 (A23187 group) or A23187 plus fresh mitochondrial suspension (1 × 10^8^/mL, Mito group), scale bar, 100 μm. (B) Representative JC‐1 fluorescence graphs of MuSCs in Ctrl, A23187 and Mito groups, red fluorescence represents the mitochondrial aggregate JC‐1 and green fluorescence indicates the monomeric JC‐1, scale bar, 100 μm. (C) Representative immunoblots and quantitative analyses of p‐Ampk, Ampk, p‐Tsc2, Tsc2, p‐mTorc1 and mTorc1 in MuSCs derived from Ctrl, A23187 and Mito groups, *n* = 3. (D) RT‐qPCR quantify the expression of mitophagy‐related genes (Bnip3, Atg5, Pink1 and Park2) in MuSCs from the Ctrl, A23187 and Mito groups, *n* = 4. (E) Representative FerroOrange fluorescence images showing intracellular Fe^2+^ accumulation of MuSCs in Ctrl, A23187 and Mito groups. Scale bar, 100 μm. (F) Representative immunoblots and quantitative analyses of ferroptosis‐associated proteins (Gpx4, Slc7a11 and Ncoa4) in MuSCs of Ctrl, A23187 and Mito groups, *n* = 3. Statistical analysis: One‐way analysis of variance test, ANOVA. Data are mean ± SEM.

To determine whether MTT interrupts the pathological cascade identified in vitro, freshly isolated donor mitochondria were locally administered into the gastrocnemius muscle beginning 6 h after sciatic nerve transection, followed by repeated injections every 3 days throughout the denervation period. At 3 days after denervation, Fluo‐4 AM staining of isolated gastrocnemius myofibres demonstrated a marked reduction in intracellular Ca^2+^ fluorescence intensity in MTT‐treated mice compared with DN_PBS controls, indicating that MTT effectively alleviated denervation‐induced Ca^2+^ overload (Figure 6A). Consistent with the restoration of calcium homeostasis, MTT markedly reduced the phosphorylation of Ampk and its downstream target Tsc2, while restoring mTorc1 phosphorylation, indicating recovery of mitophagy inhibition signal (Figure 6B). To directly assess mitophagic flux, mice received intramuscular AAV‐mt‐Keima before denervation. Three days after denervation, flow cytometric analysis demonstrated a marked increase in PE‐positive mito‐mKeima fluorescence, representing mitochondria delivered to acidic lysosomes. In contrast, MTT significantly reduced the proportion of PE‐positive mitochondria, indicating that MTT effectively suppressed excessive mitophagic flux induced by denervation (Figure 6C). Correspondingly, the expression of the mitophagy‐related proteins PINK1, Parkin and LC3B‐II was significantly reduced after MTT (Figure 6D). Consistent with the restoration of mitophagy homeostasis, ATP levels in denervated muscle were significantly increased following MTT (Figure 6E). Moreover, flow cytometric analysis demonstrated a marked increase in mitophagic flux in denervated muscle at 2 weeks, together with a corresponding reduction in FITC‐positive mitochondria in the DN_PBS group, whereas MTT significantly reduced the proportion of mitochondria undergoing lysosomal degradation (Figure S7A,B). Quantification of mean FITC fluorescence further showed that denervation resulted in a substantial loss of mitochondrial content, which was partially preserved following MTT (Figure 6C and Figure S7B). To determine whether sustained mitophagy resulted in mitochondrial loss, total mitochondrial content was quantified 4 weeks after denervation using MitoTracker Green staining followed by flow cytometric analysis. Denervated muscle exhibited a significant reduction in mean mitochondrial fluorescence intensity compared with sham controls, whereas MTT markedly preserved mitochondrial content (Figure 6F), demonstrating that suppression of excessive mitophagy prevented progressive mitochondrial loss during prolonged denervation.

7 MTT suppresses ferroptosis, preserves MuSCs and attenuates denervation‐induced muscle atrophy.

**FIGURE 6:**
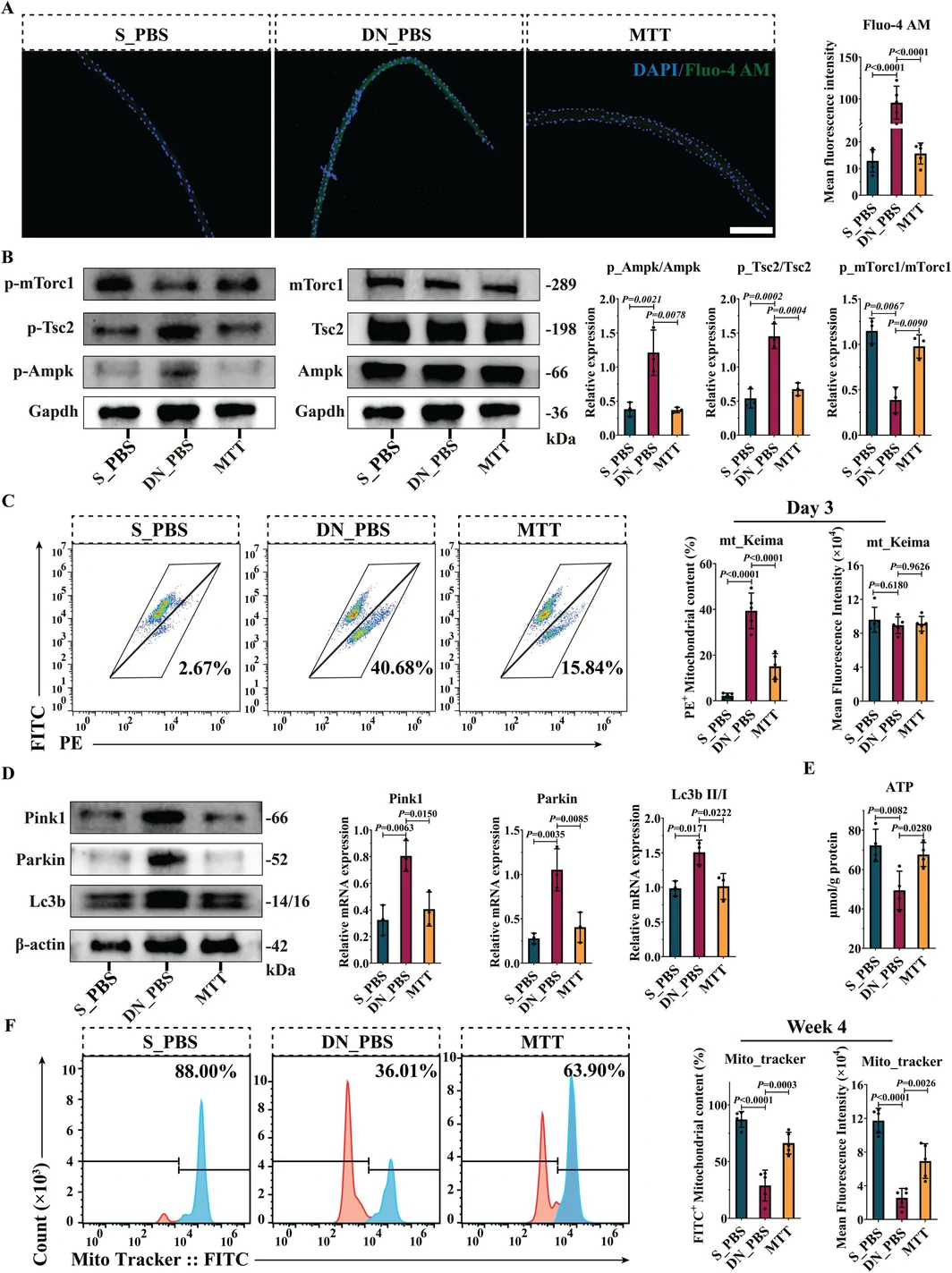
Mitochondrial transplantation restores mitochondrial homeostasis by alleviating denervation‐induced Ca^2+^ overload and excessive mitophagy. (A) Representative Fluo‐4 AM (Ca^2+^, green) staining and quantitative analysis of mean Ca^2+^ fluorescence in single gastrocnemius (Gas) fibres of mice received intramuscular injections of isolated mitochondria (MTT) or PBS 6 h after sciatic nerve transection (DN‐PBS), with sham‐operated mice as controls (S_PBS), *n* = 5 mice per group. (B) Representative immunoblots and quantitative analyses of p‐Ampk, Ampk, p‐Tsc2, Tsc2, p‐mTorc1 and mTorc1 in gastrocnemius muscle from S_PBS, DN_PBS and MTT mice. *n* = 3 mice per group. (C) Representative flow cytometric plots and quantitative analyses of mitophagic flux 3 days after denervation. The PE‐positive mitochondrial population represents mitochondria delivered to acidic lysosomal compartments and undergoing mitophagy. The mean mt‐Keima fluorescence intensity reflects the total amount of mitochondria. *n* = 5 mice per group. (D) Representative immunoblots and quantitative analyses of the mitophagy‐related proteins Pink1, Parkin and Lc3b (Lc3b‐II/I ratio) in gastrocnemius among S_PBS, DN_PBS and MTT groups, *n* = 3 mice per group. (E) ATP content in gastrocnemius muscle from S_PBS, DN_PBS and MTT mice. *n* = 4 mice per group. (F) Representative flow cytometric histograms and quantitative analyses of mitochondrial content 4 weeks after denervation in gastrocnemius muscle. The percentage of FITC‐positive mitochondria and mean fluorescence intensity are shown. *n* = 5 mice per group. Statistical analysis: One‐way analysis of variance test, ANOVA. Data are mean ± SEM.

We next examined whether inhibition of excessive mitophagy translated into suppression of ferroptotic stress and preservation of MuSC function. Compared with DN_PBS mice, MTT significantly reduced intramyofibre Fe^2+^ accumulation, lipid peroxidation and ROS generation (Figure 7A,B and Figure S8A). Although serum iron levels remained unchanged, nonheme iron accumulation within denervated muscle was significantly attenuated (Figure S8B). Furthermore, MuSCs isolated from MTT‐treated mice exhibited significantly increased expression of the ferroptosis‐protective genes Gpx4 and Slc7a11, together with reduced expression of the proferroptotic genes Facl4 and Ncoa4 (Figure S8C–F). MTT also restored the expression of the proliferation‐associated genes Cdk4, Ki67, Pcna and Ccna2, indicating preservation of MuSC proliferative capacity (Figure S8G–J). After 6 weeks of denervation, flow cytometric analysis demonstrated that MTT significantly preserved the MuSC population (Figure 7C). Histological examination further showed that MTT significantly increased the mean myofibre cross‐sectional area and markedly decreased intramuscular lipid droplet accumulation compared with DN_PBS mice (Figure 7D–F). Following delayed sciatic neurorrhaphy, MTT‐treated mice exhibited significantly greater grip strength, improved motor coordination and enhanced exercise endurance than PBS‐treated controls (Figure 7F). Collectively, these findings demonstrate that mitochondrial transplantation restores mitochondrial bioenergetics, suppresses excessive mitophagic flux, prevents progressive mitochondrial depletion, attenuates ferroptotic stress, preserves MuSC abundance and thereby partially alleviates denervation‐induced muscle atrophy while facilitating functional recovery following delayed nerve repair.

**FIGURE 7:**
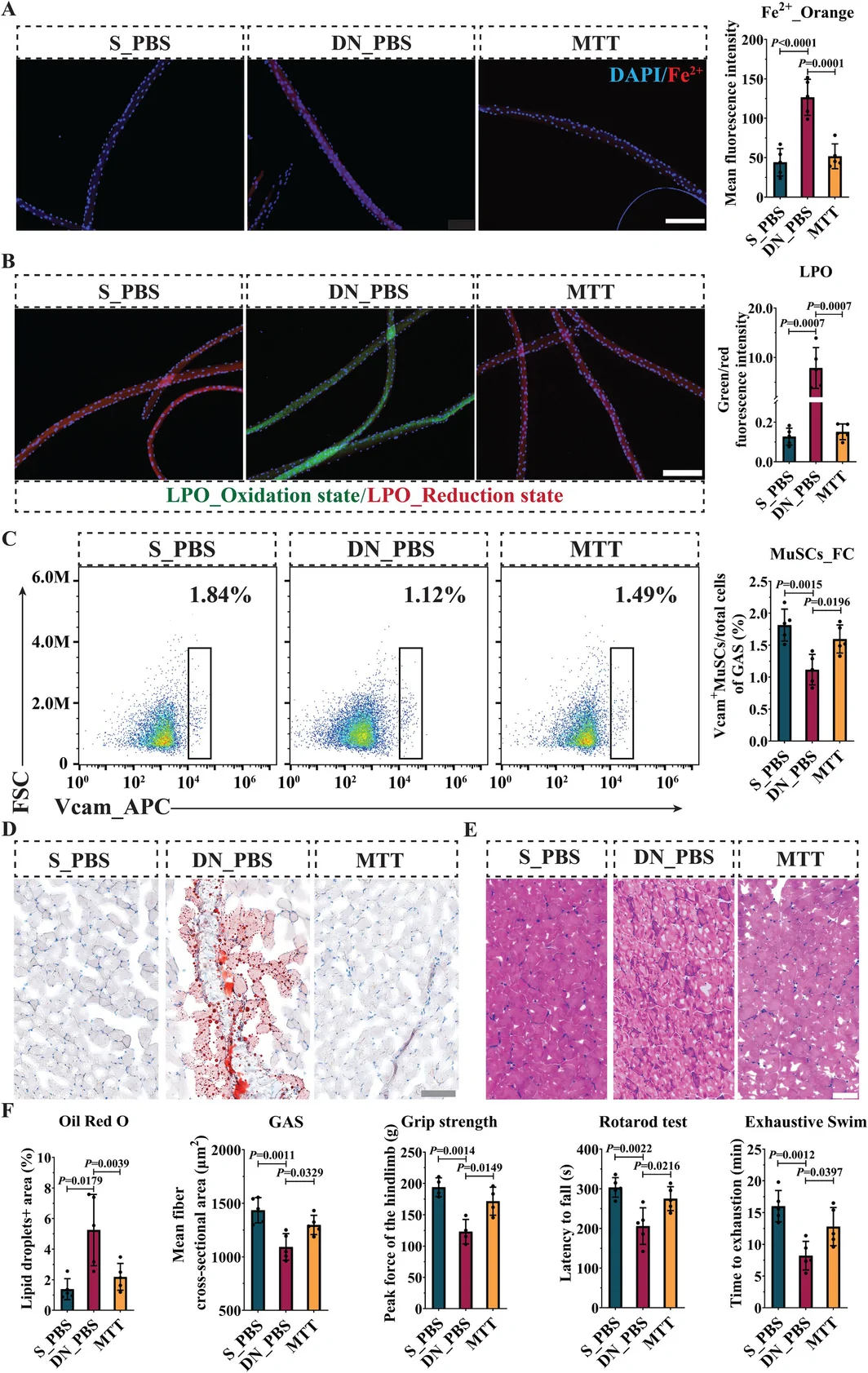
Mitochondrial transplantation attenuates ferroptotic stress, preserves MuSCs and improves muscle structure and function after denervation. (A) Representative FerroOrange fluorescence images showing intracellular Fe^2+^ accumulation in single gastrocnemius muscle fibres from S_PBS, DN_PBS and MTT mice, together with quantitative analysis of mean Fe^2+^ fluorescence intensity. Scale bar, 50 μm. (B) Representative C11‐BODIPY 581/591 fluorescence images showing the oxidation state of the lipid peroxidation probe in gastrocnemius muscle fibres. Green fluorescence represents the oxidized form of the probe, whereas red fluorescence represents the reduced form. Scale bar, 50 μm. (C) Representative flow cytometric plots and quantitative analysis of the proportion of MuSCs among total gastrocnemius muscle cells from S_PBS, DN_PBS and MTT mice after 6 weeks of denervation. *n* = 5 mice per group. (D) Representative Oil Red O staining of gastrocnemius muscle sections from S_PBS, DN_PBS and MTT mice. Scale bar, 50 μm. (E) Representative haematoxylin and eosin (H&E)‐stained transverse sections of gastrocnemius muscle from S_PBS, DN_PBS. Scale bar, 50 μm. (F) Histological and functional assessment after delayed sciatic nerve repair. Quantitative analysis of the lipid droplet‐positive area according to Figure 7D, quantitative analysis of mean myofibre cross‐sectional area according Figure 7E, peak grip strength, latency to fall in the rotarod test and time to exhaustion in the exhaustive swimming test were measured 6 weeks after sciatic neurorrhaphy. Statistical analysis: One‐way analysis of variance test, ANOVA. Data are mean ± SEM.

## 4 Discussion

Treatment of denervation‐induced muscle atrophy hinges on bridging the temporal gap between sluggish neural repair and accelerated muscle degeneration. Maintaining the largest possible MuSC pool during this interval is the key to retaining regenerative potential and achieving muscle salvage. In the present study, we identify a previously unrecognized pathogenic mechanism in which persistent Ca^2+^ overload triggers excessive mitophagic flux, mitochondrial depletion and ferroptotic stress, ultimately driving MuSC loss and progressive muscle atrophy. Importantly, MTT disrupted this pathological cascade, preserved MuSC abundance, partially alleviated muscle degeneration and improved functional recovery after delayed nerve repair (Figure 8). These findings provide a mechanistic framework explaining how mitochondrial dysfunction accelerates denervation‐induced muscle deterioration and suggest MTT as a potential bridging strategy during the interval between nerve injury and reconstruction.

**FIGURE 8:**
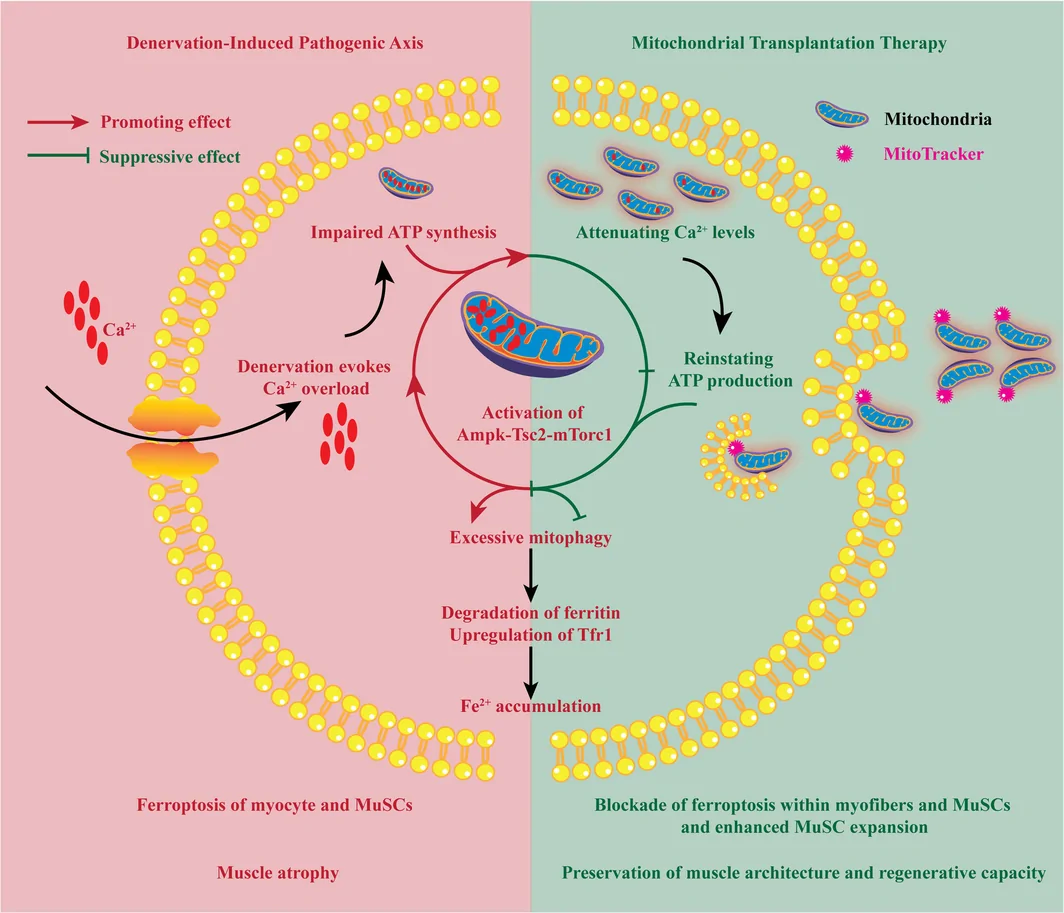
MTT preserves MuSCs by disrupting a Ca^2+^ overload‐mitophagy positive‐feedback loop to suppress ferroptosis, thereby alleviating denervation‐induced atrophy Left (Red): Pathogenic cascade leading to MuSC depletion and muscle atrophy. Denervation‐induced persistent Ca^2+^ overload compromises mitochondrial bioenergetics, resulting in impaired ATP production and activation of the Ampk–Tsc2–mTorc1 pathway. This signalling imbalance promotes excessive mitophagic flux, leading to progressive mitochondrial loss. The resulting loss of mitochondrial Ca^2+^‐buffering capacity and ATP‐generating capacity further aggravates Ca^2+^ overload and mitochondrial dysfunction, thereby establishing a self‐amplifying positive‐feedback loop. Sustained mitochondrial quality‐control stress is accompanied by iron dysregulation, lipid peroxidation and reactive oxygen species accumulation, promoting ferroptotic stress in MuSCs. This pathological cascade contributes to MuSC depletion, impaired proliferative potential and progressive denervation‐induced muscle atrophy. Right (Green): Therapeutic rescue by mitochondrial transplantation therapy (MTT). Intramuscular transplantation of functional mitochondria provides healthy organelles that are internalized by MuSCs and restore mitochondrial bioenergetics. By buffering pathological Ca^2+^ overload and improving ATP production, MTT disrupts the self‐amplifying cycle of Ca^2+^ overload and excessive mitophagy. MTT suppresses excessive mitophagic flux, preserves mitochondrial content and attenuates iron‐dependent lipid peroxidation and ferroptotic stress. Consequently, MTT preserves the MuSC pool and its proliferative potential, partially maintains myofibre integrity and facilitates functional recovery following delayed nerve repair. These findings support MTT as a potential bridging strategy to preserve the regenerative competence of denervated muscle during the interval before successful reinnervation. Red arrows/borders depict the degenerative pathway in denervated muscle; green arrows/borders depict the restorative effects of MTT.

Classically, denervation‐induced myoatrophy was ascribed to heightened ubiquitin‐proteasome activity and wholesale proteolysis of contractile proteins. Our temporal transcriptomic analysis revealed that although proteolytic pathways were activated early after denervation, signatures associated with oxidative stress, mitochondrial dysfunction, cell death and impaired proliferation became increasingly prominent during later stages. These findings support the concept that MuSC exhaustion, rather than protein degradation alone, represents a critical determinant of progressive muscle failure. Consistent with previous studies demonstrating that dysfunctional MuSC dynamics contribute to muscle wasting [14, 15, 29, 30], our work extends this concept by identifying a mitochondrial quality‐control pathway that directly links early denervation stress to MuSC ferroptosis.

Ca^2+^ dyshomeostasis has long been implicated in denervation pathology [31]; however, its downstream consequences on mitochondrial fidelity and cell fate remained poorly defined. Skeletal muscle is the organ with the largest Ca^2+^ storage capacity [32]. In the denervated state, persistent depolarization drives a sustained Ca^2+^ rise that exceeds mitochondrial buffering capacity, collapsing mitochondrial membrane potential and curtailing ATP synthesis [33]. Perturbed energy metabolism activates the Ampk‐Tsc2‐mTorc1 signalling axis, thereby initiating mitophagy. In contrast to a transient protective role, persistent mitophagy in denervated muscle progressively depletes the mitochondrial pool, which in turn aggravates cytosolic Ca^2+^ overload and bioenergetic failure, establishing a self‐perpetuating vicious cycle [34]. Moreover, this self‐amplifying Ca^2+^ overload‐mitophagy loop disrupts cellular iron metabolism, thereby predisposing cells to ferroptosis. Importantly, our findings do not imply that mitophagy is the sole determinant of denervation‐induced muscle atrophy. Instead, excessive mitophagic flux represents a major contributor within a broader pathological network that also includes loss of neural trophic support, mechanical unloading, impaired excitation‐contraction coupling, fibrosis and activation of other catabolic pathways. Indeed, the observation that suppression of excessive mitophagy attenuated but did not completely prevent muscle atrophy indicates that maintenance of muscle mass requires both mitochondrial preservation and restoration of physiological neural input.

In addition, previous studies have implicated mitochondria‐mediated apoptosis in denervated skeletal muscle [35, 36]. Because these investigations preceded the recognition of ferroptosis as a regulated form of cell death, the potential contribution of ferroptosis could not be evaluated [37]. Given that mitochondrial dysfunction, oxidative stress and disrupted iron homeostasis are shared features of both apoptosis and ferroptosis, it is plausible that these pathways coexist or interact during denervation‐induced muscle degeneration [38]. Although the present study focused specifically on ferroptosis in MuSCs, future studies are warranted to define the relative contribution and potential crosstalk between apoptotic and ferroptotic signalling during denervation.

The therapeutic implications of this mechanism are substantial. Relevant studies have shown that administration of Ca^2+^ chelators such as Bapta‐am within 1 week after nerve injury effectively attenuates cellular Ca^2+^ overload and mitigates the loss of skeletal muscle wet weight [27]. However, both systemic and local administration risk perturbing physiological Ca^2+^ signalling, disrupting iron/zinc homeostasis and suppressing haematopoiesis while compromising immune function. Confronted with this intricate pathological network, MTT has emerged as a novel intervention, exerting protection in cardiac ischemia and stroke paradigms [39]. In contrast to administration of Ca^2+^ chelators, MTT offers superior flexibility in mechanistic scope, therapeutic time‐window, dosing latitude and translational feasibility. Moreover, the first‐in‐human trials of mitochondrial transfer in cardiac and neurological disorders have established preliminary safety and efficacy, paving the way for its application in neuromuscular rehabilitation [40]. Nevertheless, key challenges remain, including optimizing mitochondrial delivery efficiency, developing sustained release carriers such as hydrogel systems, and validating efficacy in chronic atrophy models where fibrosis may limit engraftment. In addition, several limitations should be acknowledged. First, our pharmacological approach using Mdivi‐1 should be interpreted cautiously because Mdivi‐1 primarily targets Drp1‐dependent mitochondrial fission rather than serving as a specific mitophagy inhibitor. Therefore, additional genetic models will be required to further define the specific contribution of mitophagy. Second, MTT was initiated shortly after denervation; whether mitochondrial transplantation remains effective after established chronic atrophy requires further investigation. Finally, although MTT partially preserved muscle structure and function, it cannot replace neural reconstruction. Long‐term muscle maintenance ultimately depends on restoration of neural input and mechanical activity.

In conclusion, this study redefines denervation‐induced muscle atrophy as a disorder of mitochondrial quality control and regenerative capacity rather than a consequence of proteolysis alone. By identifying the persistent Ca^2+^ overload—excessive mitophagy‐ferroptosis axis, we establish a mechanistic pathway linking early denervation events to irreversible muscle decline. Mitochondrial transplantation interrupts this pathological circuit and preserves the regenerative competence of denervated muscle, suggesting that mitochondrial therapy may serve as a clinically relevant bridge strategy to extend the therapeutic window before successful nerve reconstruction.

## Ethics Statement

All animal experiments were approved by the Fuzhou University Affiliated Provincial Hospital Experimental Animal Welfare and Ethics Committee (approval no. IACUC‐FPH‐SL‐20250527[0943]).

## Conflicts of Interest

The authors declare no conflicts of interest.

## Supporting information


**Figure S1:** Denervated skeletal muscle exhibits Ca^2^⁺ dysregulation, enhanced autophagy and ferroptosis. (A) The volcano plot illustrates DEGs in skeletal muscle from DN and Sham; calcium‐metabolism genes are highlighted in blue, autophagy genes in red. (B–D) GO enrichment analysis of biological process (GO_BP, B), molecular function (GO_MF, C) and cellular component (GO_CC, D) for upregulated DEGs and downregulated DEGs. Dot size indicates gene count; colour intensity indicates ‐log10(*p*‐value). (E) Quantification of mean Ca^2^⁺ fluorescence intensity in isolated gastrocnemius (Gas) fibres of Sham versus DN mice, *n* = 5 mice per group. (F) As assessed in Figure 1D, the abundance of damaged mitochondria in Gas TEM sections was quantified and compared between Sham and DN groups, *n* = 5 mice per group. (G) Quantitative comparison of serum iron levels and Gas nonheme iron content between Sham and DN mice. *n* = 5 mice per group. (H) As assessed in Figure 1E, quantification of Fe^2+^ fluorescence in isolated Gas fibres from DN and Sham mice, *n* = 5 mice per group. (I) As determined from Figure 1F, the Oil Red O‐positive area in Gas cryosections was calculated and compared between Sham and DN mice, *n* = 5 mice per group. (J) RT‐qPCR analysis of ferroptosis‐related gene expression (Gpx4, Slc7a11, Facl4 and Ncoa4) in Gas muscle from Sham and DN mice, *n* = 4 mice per group. Statistical analysis: Two‐sided unpaired *t*‐test. Data are presented as mean ± SEM.
**Figure S2:** Denervation impairs muscle satellite cell (MuSC) proliferation by 2 weeks (A) The gating strategy for flow cytometric analysis or sorting of MuSCs from mouse skeletal muscle, H, high expression; L, low expression. (B and C) According to Figure 2D and E, statistical analysis of the proportion of MuSCs in gastrocnemius (Gas) between Sham and denervated (DN) mice at 2 weeks (B) or 4 weeks (C) postsurgery, *n* = 5 mice per group. (D) As assessed in Figure 2F, the number of Pax7^+^ MuSCs in Gas muscle was calculated between Sham and DN mice, *n* = 5 mice per group. (E) The changes in the number of MuSCs in the Gas of mice at 2, 4, 6 and 8 weeks after denervation, presented as a proportion of total cells, *n* = 5 mice per group. (F) Statistical analysis of the difference in wet weight of Gas muscle between Sham and DN groups, *n* = 5 mice per group. (G) Representative immunofluorescence images of neuromuscular junctions in Gas muscle of Sham and DN mice, scale bar, 100 μm. (H) Representative immunofluorescence micrographs and statistical analysis of Type I and II fibre in Gas muscle, comparing Sham versus DN mice, *n* = 5 mice per group. (I) Representative haematoxylin and eosin (H&E) staining images of gastrocnemius (Gas) muscle sections from Sham and DN mice, with quantitative analyses of myofibre density, mean myofibre cross‐sectional area (CSA) and myofibre diameter distribution. Scale bar, 100 μm. Statistical analysis: (A–D, F, H and myofibre density and mean CSA in I) two‐sided unpaired Student's *t*‐test; (myofibre diameter distribution in I) two‐way ANOVA. Data are presented as mean ± SEM.
**Figure S3:** Ca^2+^ overload induces Mitophagy in MuSCs via the Ampk‐Tsc2‐mTorc1 axis (A) According to Figure B, statistical analysis of the expression of Ampk, p‐Ampk, Tsc2, p‐Tsc2, mTorc1 and p‐mTorc1 in Gas of Sham and DN mice, *n* = 3 biological replicates. (B) According to Figure D, statistical analysis of the difference in mean Ca^2+^ fluorescence intensity among MuSCs in Ctrl, A23187, and Bapta‐am groups, *n* = 4. (C) According to Figure E, quantitative analysis of the green/red fluorescence intensity ratio (%) in MuSCs from Ctrl, A23187 and Bapta_am groups, *n* = 5. (D) Difference in ATP production level of MuSCs among Ctrl, A23187 and Bapta_am groups, *n* = 4. (E) RT‐qPCR quantifies the expression differences of mitophagy genes (Bnip3, Atg5, Pink1 and Park2) in MuSCs among Ctrl, A23187 and Bapta_am groups, *n* = 4. Statistical analysis: One‐way analysis of variance test, ANOVA. Data are presented as mean ± SEM.
**Figure S4:** Excessive mitophagy aggravates Fe^2+^ accumulation and triggers ferroptosis (A) According to Figure 4A, quantitative analysis of Fe^2+^‐metabolism‐related proteins (Fth1 and Tfr1) in Gas muscle of Sham and DN groups, *n* = 3. (B) Representative DCFH‐DA staining for reactive oxygen species (ROS) in isolated Gas myofibres and statistical analysis of mean ROS fluorescence intensity in single myofibres of Sham and DN groups, scale bar,100 μm, *n* = 5. (C) According to Figure 4E, statistical analysis of the differences in the proportion of ROS⁺ MuSCs among Ctrl, A23187 and Mdivi‐1 groups. (D) Representative Fe^2+^ staining and statistical analysis of mean fluorescence intensity in MuSCs treated with vehicle (Ctrl group), 5‐μM A23187 (A23187 group) or A23187 plus 5‐μM Mdivi‐1 (Mdivi‐1 group). Scale bar, 50 μm, *n* = 5. (E) According to Figure 4F, quantitative analysis of green/red fluorescence intensity (%) in MuSCs from Ctrl, A23187 and Mdivi‐1 groups, red fluorescence represents the cellular aggregate LPO and green fluorescence indicates the monomeric LPO, *n* = 5. (F) Immunoblots and statistical analysis of the expression of ferroptosis‐related proteins (Gpx4, Slc7a11 and Ncoa4) in MuSCs among Ctrl, A23187 and Mdivi‐1 groups, *n* = 3. (G) RT‐qPCR quantifies the expression of Pink1 in MuSCs treated with scrambled control siRNA (Scr) or Pink1‐targeting siRNA (siPink1), *n* = 5. (H) Immunoblots and statistical analysis of the expression of Fe^2+^‐metabolism‐related proteins (Fth1 and Tfr1) in MuSCs among Scr_Ctrl, Scr_A23187, siPink1_Ctrl and siPink1_A23187 groups. (I) Representative Fe^2+^ staining and statistical analysis of mean Fe^2^⁺ fluorescence intensity in MuSCs among Scr_Ctrl, Scr_A23187, siPink1_Ctrl and siPink1_A23187 groups, scale bar, 50 μm, *n* = 5. (J) Immunoblots and statistical analysis of the expression of ferroptosis markers (Gpx4, Slc7a11 and Ncoa4) in MuSCs among Scr_Ctrl, Scr_A23187, siPink1_Ctrl and siPink1_A23187 groups, *n* = 3. Statistical analysis: (A, B and G) Two‐sided unpaired *t*‐test, (C‐F and H–J) One‐way analysis of variance test, ANOVA. Data are presented as mean ± SEM.
**Figure S5:** Experimental design for the application of mitochondrial transplantation therapy (MTT) in vivo and in vitro. (A) Schematic diagram of mitochondrial isolation, purification and transplantation. (B) Representative micrographs of MitoTracker‐stained mitochondria, scale bar, 100 μm. (C) Representative fluorescence image of in vivo mitochondrial transplantation; red: MitoTracker deep red FM, green: *α*‐Tubulin, scale bar, 100 μm. (D) Representative image of mitochondrial uptake after ex vivo mitochondrial transplantation; red: MitoTracker deep red FM, scale bar, 100 μm.
**Figure S6:** Mitochondrial transplantation mitigates Ca^2^⁺ overload‐induced mitophagy to suppress ferroptosis in MuSCs. (A) According to Figure 5A, comparison of mean Ca^2^⁺ fluorescence intensity in MuSCs among Ctrl, A23187 and Mito groups, *n* = 4. (B) According to Figure 5B, statistical analysis of JC‐1 in MuSCs from Ctrl, A23187 and Mito groups, expressed as green/red fluorescence intensity (%), *n* = 6. (C) Differences in ATP production of MuSCs among the Ctrl, A23187, and Mito groups, *n* = 5. (D) According to Figure 5E, statistical analysis of mean fluorescence differences of Fe^2+^ in MuSCs among the Ctrl, A23187 and Mito groups, *n* = 5. (E) Representative ROS fluorescence staining and statistical analysis of mean fluorescence intensity of ROS in Ctrl, A23187 and Mito groups MuSCs, scale bar, 100 μm, *n* = 5. (F) Representative flow cytometric histograms and statistical analysis of the differences in the proportion of ROS^+^ MuSCs in the Ctrl, A23187 and Mito groups, *n* = 5. (G) Representative LPO fluorescence staining and statistical analysis of green/red fluorescence intensity (%) in MuSCs from Ctrl, A23187 and Mito groups, red fluorescence indicates nonoxidized lipids, green fluorescence indicates lipid hydroperoxides, scale bar, 100 μm, *n* = 4. Statistical analysis: One‐way analysis of variance test, ANOVA. Mean ± SEM.
**Figure S7:** Flow cytometric analysis of mitophagy flux using mt‐Keima reporter after denervation injury. (A) Representative gating strategy for flow cytometric quantification of mitophagy flux using mt‐Keima fluorescence. Cells were sequentially gated based on forward and side scatter (FSC/SSC) to identify target cells, followed by FSC‐height/FSC‐area analysis to exclude doublets and select single cells. Mitophagy flux was determined by analysing mt‐Keima fluorescence signals in the PE and FITC channels, representing mitochondria exposed to acidic lysosomal environments and mitochondria maintained in nonacidic compartments, respectively. (B) Representative mt‐Keima flow cytometry plots and quantitative analysis of mitochondrial autophagy flux at 2 weeks after denervation. The PE‐positive mitochondrial population represents mitochondria localized within acidic autolysosomal compartments and indicates active mitophagic flux. The FITC mean fluorescence intensity of mito‐mKeima‐labelled mitochondria was analysed to estimate the preserved mitochondrial pool. *n* = 5 mice per group. Statistical analysis: One‐way analysis of variance test, ANOVA. Data are presented as mean ± SEM.
**Figure S8:** MTT attenuates denervation‐induced ferroptotic responses and restores muscle regenerative activity. (A) Representative ROS fluorescence staining and statistical analysis of ROS fluorescence intensity in single myofibres of Gas among S_PBS, DN_PBS and MTT groups, scale bar, 40 μm, *n* = 5 mice per group. (B) Statistical analysis of serum iron levels and Gas nonheme iron content between the S_PBS, DN_PBS and MTT groups, *n* = 5 mice per group. (C–F) RT‐qPCR quantified the expression of ferroptosis‐related genes (Gpx4, Slc7a11, Facl4 and Ncoa4) in Gas of the S_PBS, DN_PBS and MTT mice, *n* = 5 mice per group. (G–J) RT‐qPCR quantified the expression of cell proliferation‐related genes (Cdk4, ki67, Pcna and Ccna2) in Gas of the S_PBS, DN_PBS and MTT mice, *n* = 5 mice per group. Statistical analysis: One‐way analysis of variance test, ANOVA. Data are presented as mean ± SEM.

## Data Availability

The data that support the findings of this study are available from the corresponding author upon reasonable request.
