# Supplementary material for: Mitochondrial Transplantation Preserves Regenerative Capacity of Denervated Skeletal Muscle by Disrupting a Ca2+ Overload‐Mitophagy‐Ferroptosis Circuit
Source: J Cachexia Sarcopenia Muscle. 2026 Nov 5;17(6):e70416. doi: 10.1002/jcsm.70416 (PMC13735212; doi:10.1002/jcsm.70416)
Supplement: Supplementary file 1 [file JCSM-17-e70416-s001.docx]

Supplementary materials

**Mitochondrial Transplantation Preserves Regenerative Capacity of Denervated Skeletal Muscle by Disrupting a Ca²⁺ Overload- Mitophagy- Ferroptosis Circuit**

***Additional methods***

1. **Isolation and Culture of Murine Skeletal Muscle Satellite Cells (MuSCs)**

Primary satellite-cell isolation and culture were performed as previously described^1–3^. Briefly, 1-week-old mice were euthanized with CO₂ and disinfected by immersion in 75 % ethanol for 3 min. In a biosafety hood, all skeletal muscles were dissected, finely minced, and cleared of adipose and connective tissues. Muscle was incubated in digestion solution (Dulbecco's Modified Eagle Medium high glucose (DMEM) with 3 % P/S, 1.2 mg ml⁻¹ Dispase II, and 2 mg ml⁻¹ Collagenase IV) for 1 h at 37 °C, pipetted every 20 min. Digestion was quenched with DMEM containing 10 % FBS and 1 % antibiotic–antimycotic. Cells were passed through a 70 μm strainer, spun at 1 500 g for 5 min, washed twice in DMEM/20 % FBS/1 % P/S, and re-suspended. Cells derived from five mice were seeded per 10 cm dish and cultured at 37 °C, 5 % CO₂ in a Thermo incubator. Over the following 3 days, floating satellite cells were collected daily via pre-plating and re-seeded for expansion. Proliferation medium consisted of Ham's F-10 supplemented with 10 % FBS, 1 % antibiotics, 5 ng ml⁻¹ bFGF, 5 ng ml⁻¹ IL-1α, 5 ng ml⁻¹ IL-13, 10 ng ml⁻¹ IFN-γ, and 10 ng ml⁻¹ TNF-α.

2. **Small interfering RNA (siRNA) transfection of MuSCs**

Pink1 knockdown in MuSCs was performed using small interfering RNA (siRNA) targeting mouse Pink1. MuSCs were seeded 24 h before transfection and transfected at approximately 50% confluence using Lipofectamine™ 2000 (Invitrogen, USA) according to the manufacturer’s instructions. The final concentration of siPink1 was 30 nM. A scrambled siRNA was used as the negative control (Scr). The sequences of siPink1 were as follows: forward, 5′-GUCAGGAGAUCCAGGCAAUUUTT-3′; reverse, 5′-AAAUUGCCUGGAUCUCCUGACTT-3′.

3. **Mitochondrial labeling and extraction**

Mitochondrial labeling and extraction were performed following previously described protocols^4^. Primary MuSCs were seeded on 0.2 % Matrigel-coated 10 cm dishes at 1 × 10⁶ cells/dish and cultured to 70 % confluence in growth medium. Subsequently, the medium was then switched to a differentiation-inducing medium supplemented with 2% horse serum. Muscle satellite cells (MuSCs) that had undergone differentiation for 3 to 5 days were harvested for mitochondrial isolation. To fluorescently tag functional mitochondria, cells were incubated with Mito-Tracker Red CMXRos (100 nM, Beyotime, C1049B) in phenol-red-free F-10. After two washes with warm PBS, fresh medium was added for 15 min chase to allow dye retention in polarized mitochondria. Labeled cells were detached with Trypsin (0.1%, 37 °C, 3 min), pooled (≈4 × 10⁷ cells per isolation), and processed with the Minute™ Mitochondria Isolation Kit (Invent Biotechnologies, MP-007) at 4 °C. Briefly, cells were washed once with ice-cold PBS, resuspended in 250 µL Buffer A (supplemented with 1× protease/phosphatase inhibitors), and incubated on ice for 5 min. The suspension was transferred into a pre-chilled centrifuge column and centrifuged at 16 000 × g for 30 s. The pellet was re-suspended in 250 µL Buffer A, passed through the same column once more to increase yield, and centrifuged again. The combined effluent was mixed with 300 µL Buffer B (1:1.2 v/v), vortexed, and centrifuged at 16 000 × g for 10 min to remove cytosolic contaminants. The crude organelle pellet was washed with 200 µL Buffer B (8 000 × g, 5 min) and finally re-suspended in 200 µL ice-cold PBS. Intact labeled mitochondria were pelleted at 16 000 × g for 15 min, quantified by BCA, and kept on ice until transplantation (within 2 h).

4. **Transcriptome data acquisition and bioinformatics analysis**

Raw RNA-sequencing (RNA-seq) count matrices and corresponding metadata were downloaded from the NCBI Gene Expression Omnibus (GEO) under accession number GSE44205 using the Bioconductor package GEOquery (v2.62.2). Samples were retained only if they met the following criteria: (i) species = Mus musculus, (ii) RNA-seq strategy = paired-end, and (iii) library source = transcriptome. Low-quality samples with <5 million mapped reads or a ribosomal RNA ratio >5 % were excluded. After import into R v4.3.2, gene-level counts were converted to a DESeqDataSet object by the DESeq2 package (v1.38.3). Pre-filtering removed genes with <10 counts across all samples. The dataset was normalized with the DESeq2 median-of-ratios method, and blind size-factor estimation was performed prior to differential expression. Differential expression analysis between denervated wild type (n = 3) and control wild type (n = 3) groups was performed using DESeq2 with the Wald test and a false-discovery rate (FDR) threshold of 5 % (Benjamini–Hochberg). Genes with an absolute log2-fold change ≥1 and a *P* value <0.05 were considered significantly differentially expressed. Volcano plots and heat-maps were generated with ggplot2 (v3.4.4) and pheatmap (v1.0.12), respectively. Gene Ontology (GO) and Kyoto Encyclopedia of Genes and Genomes (KEGG) pathway enrichment analyses of differentially expressed genes were carried out with the clusterProfiler package (v4.8.2). Background correction used the org.Mm.eg.db annotation database (v3.16.0). Only terms with an FDR <0.05 were regarded as significantly enriched.

5. **Myofibre isolation**

Single myofibres were isolated from EDL muscle following the method as described before^5,6^. Briefly, EDL muscle was isolated and incubated with digestion medium containing 0.2% collagenase for 75 min. After digestion, myofibres were transferred to a horse serum-coated 24-well plate and gently washed for three times with the washing medium (DMEM supplemented with 10% FBS and 1% penicillin and streptavidin). In each well, ~20 myofibres were washed for three times before being replaced with culture medium (DMEM supplemented with 20% FBS, 1% penicillin–streptomycin, and 1% chicken embryo extract (C19041654, USBiological, Salem, MA).

6. **Histological analysis of skeletal muscle**

Immediately after euthanasia, the gastrocnemius muscle was carefully dissected from the hind-limb, and rapidly frozen in isopentane pre-cooled in liquid nitrogen (−160 °C) for 30 s. Tissues were stored at −80 °C until sectioning. Ten-micrometer transverse cryosections were obtained at −20 °C (Leica CM3050S) and mounted on adhesive glass slides (SuperFrost Plus, Thermo Fisher). For hematoxylin and eosin (H&E) staining, sections were air-dried for 30 min, fixed in 4 % (w/v) paraformaldehyde (PFA) for 10 min, rinsed in PBS, and then processed with Mayer’s hematoxylin (5 min), tap-water bluing (3 min), eosin Y (1 min), and graded ethanol dehydration (70 %, 95 %, 100 %, each 1 min). Slides were cleared in xylene and mounted with neutral balsam. Myofiber cross-sectional area (CSA) was quantified from ≥5 random fields per mouse using ImageJ (v1.54g) with a blinded observer; a minimum of 300 fibers per animal were analyzed.

For Oil Red O (ORO) staining of neutral lipids, frozen sections were air-dried and fixed in 4 % PFA (10 min). After three PBS washes, sections were incubated in 0.5 % (w/v) ORO working solution (prepared fresh by diluting 0.5 g ORO in 100 ml 60 % triethyl-phosphate) for 15 min at room temperature, differentiated in 60 % triethyl-phosphate (2 × 30 s), and washed in running tap water (2 min). Nuclei were counter-stained with Mayer’s hematoxylin (30 s), and sections were mounted with aqueous glycerol jelly. Quantification was performed by calculating the percentage ORO-positive area (red pixels) relative to total tissue area using color-threshold segmentation in ImageJ; five non-overlapping fields per section, three sections per mouse, were averaged.

7. **Immunofluorescence staining**

Single myofibers and in-vitro-treated MuSCs were labeled with fluorescent probes for Ca²⁺, iron, ROS, mitochondrial membrane potential (JC-1), and lipid peroxidation. For single-myofiber assays, a minimum of 100 myofibers per sample were processed, and all staining steps were completed within 2 hours post-isolation to preserve viability. For intracellular Ca²⁺ imaging, fibers/MuSCs were loaded with Fluo-4 AM (2 μM; Beyotime, S1060) in 0.1 M PBS for 20 min at 37 °C, followed by a 20-min de-esterification period. Fe²⁺ levels were assessed using FerroOrange (1:1000; Dojindo, F374) incubated for 30 min at 37 °C in darkness. ROS production was detected with Reactive Oxygen Species Assay Kit (1:5000; Solarbio, CA1410/CA1420) incubated for 30 min at 37 °C. To visualize changes in mitochondrial membrane potential (ΔΨm) of MuSCs, cells were incubated with the JC-1 fluorescent probe (1:200, Beyotime, C2006) for 20 min at 37 °C, 5 % CO₂ in the dark. Lipid peroxidation (LPO) was visualized using C11-BODIPY 581/591 (2 μM; Beyotime, S0043S) incubated for 30 min at 37 °C, which shifts from red to green fluorescence upon oxidation. After staining, all fibers were washed three times with fresh 0.1 M PBS and immediately imaged using a fluorescence microscope.

For immunofluorescence staining of skeletal muscle tissue, after euthanasia, the gastrocnemius was dissected, and immediately fixed in 4 % (w/v) paraformaldehyde (PFA) in 0.1 M PBS (pH 7.4) for 2 h at 4 °C. Fixed muscles were sequentially cryoprotected in 15 % sucrose (4 °C, 4 h) and 30 % sucrose (4 °C, overnight), then embedded in OCT compound (Tissue-Tek) and rapidly frozen in isopentane pre-cooled in liquid nitrogen. 10-µm transverse cryosections were cut at −20 °C (Leica CM3050S), collected on adhesive glass slides (SuperFrost Plus), and stored at −80 °C until use. Before staining, sections were air-dried for 30 min at RT, re-hydrated in PBS (3 × 5 min), and subjected to antigen retrieval by incubation in 0.01 M sodium citrate buffer (pH 6.0) at 95 °C for 10 min. After cooling to RT, sections were permeabilized with 0.3 % Triton X-100 in PBS (15 min) and blocked for 1 h in blocking buffer: 5 % (v/v) normal goat serum + 2 % (w/v) BSA in PBS. Primary antibodies (anti-Pax7 (1:50, Developmental Studies Hybridoma Bank, AB_528428); anti-MyoD (1:50, ABclonal, A0671); anti-Myh1 (1:500, Servicebio, GB112130-100); anti-Myh7 (1:500, Servicebio, GB112131-100); anti-NF200 (1:200, Proteintech, 18934-1-AP))were diluted in blocking buffer and applied overnight at 4 °C. Sections were then washed in PBS and incubated with species-appropriate Alexa Fluor-conjugated secondary antibodies (1:500; Invitrogen) for 1 h at RT in the dark. For neuromuscular-junction (NMJ) staining in skeletal muscle, α-bungarotoxin conjugated to Alexa Fluor ® 488 (α-BTX, 1:1000; ThermoFisher) was included in the secondary-antibody cocktail to label postsynaptic acetylcholine receptors. After final washes, nuclei were counter-stained with DAPI (1 µg ml⁻¹, 5 min) and slides mounted with Prolong Diamond antifade medium (Thermo Fisher).

8. **Transmission electron microscope**

Gastrocnemius fragments (1 mm³) were fixed in 2.5 % glutaraldehyde at 4 °C for 4 h, post-fixed with 1 % OsO₄ for 2 h, dehydrated, and embedded in Epon-812. Ultrathin sections (60 nm) were stained with 2 % uranyl acetate (10 min) and lead citrate (5 min), then viewed at 80 kV (JEM-1400Flash). Mitochondrial injury was defined as swollen organelles with fractured/absent cristae or ruptured outer membranes. Mitophagy was identified as mitochondria within double-membrane autophagosomes or mitophagosomes. Ferroptosis morphology comprised shrunken mitochondria with condensed, reduced cristae, intact but wrinkled outer membranes, and no apoptotic bodies. Ten random fields per mouse were analyzed blind using ImageJ to measure % of damaged or engulfed organelles.

9. **Flow cytometry**

MuSCs were FACS-isolated/analysed from the gastrocnemius-soleus complex following published protocols^7^. Specifically, hind-limb muscles of euthanized mice were excised, minced, and cleared of fat and connective tissue, then digested for ~40 min at 37 °C in DMEM containing 3 % P/S, 1.2 mg ml⁻¹ Dispase II and 2 mg ml⁻¹ Collagenase IV with trituration every 15 min; the reaction was stopped by addition of FBS. Cells were filtered (70 μm) and pelleted by centrifugation at 1 500 g for 5 min. The cells were re-suspended in FACS buffer, incubated with PerCP/Cy5.5-CD45/CD11b/CD31 (Elabscience, E-AB-F1136J/E-AB-F1081J/ E-AB-F1180J), PE-Sca1 (Elabscience, E-AB-F1191D)和APC-Vcam (Elabscience, E-AB-F1091E) for 45 min on ice. Cells were washed with FACS buffer, re-centrifuged, and re-suspended in FACS buffer. CD45⁻CD11b⁻CD31⁻Sca1⁻Vcam⁺ MuSCs were sorted on a BD FACSAriaIII. The proportion of CD45⁻CD11b⁻CD31⁻Sca1⁻Vcam⁺ MuSCs was quantified with a BD Accuri^TM^ C6.

For FACS analysis of in-vitro expanded MuSCs, cells were grown to 70–80 % confluence on Matrigel-coated 6-well plates, rinsed with PBS, and dissociated with 0.1 % trypsin at 37 °C for 3 min. Trypsinization was halted by adding DMEM containing 10 % FBS, followed by centrifugation at 1 000 g for 5 min and re-suspension in ice-cold FACS buffer. Single-cell suspension was filtered (40 µm), counted, and aliquoted at 0.5-1 × 10⁶ cells per tube. The level of ROS was evaluated by using the fluorescence intensity of 2’-7’-dichloroflorescein (DCFH-DA, Solarbio, CA1410). The cells were incubated with DMEM medium containing 20 μmol/L DCFH-DA for 30 min at 37 °C in the dark. After rinsing, digesting and filtration, the cells were analyzed within 1 h. Samples were analyzed on a BD C6, at least 10000 events within the singlet gate were recorded per sample. Data were analyzed with FlowJo v10.9; results are presented as percentage positive population.

For mitophagic flux assay, mice received intramuscular injection of AAV-CMV-4×mito-mKeima-red-WPRE-pA (BrainVTA, PT-12804) into the gastrocnemius muscle 3 weeks before sciatic nerve transection to enable stable mitochondrial expression of mt-Keima^8^. Gastrocnemius muscles were harvested 3 days or 2 weeks after denervation, enzymatically dissociated into single-cell suspensions, and analyzed by flow cytometry. The mt-Keima fluorophore exhibits pH-dependent excitation spectra, allowing discrimination between mitochondria residing in the neutral cytoplasm and those delivered to acidic lysosomes. FITC-positive fluorescence represented mitochondria remaining outside lysosomes, whereas PE-positive fluorescence represented mitochondria localized within lysosomes. Mitophagic flux was quantified as the proportion of PE-positive mt-Keima fluorescence.

Total mitochondrial content was assessed 4 weeks after denervation using MitoTracker™ Green FM (Invitrogen, M46750). Gastrocnemius muscles were enzymatically dissociated into single-cell suspensions and incubated with MitoTracker Green according to the manufacturer's instructions. Mean fluorescence intensity was analyzed by flow cytometry and used as an indicator of total mitochondrial mass.

10. **Iron assay**

Total iron content was quantified using the Solarbio Tissue Iron Colorimetric Assay Kit (BC4355, Beijing, China) according to the manufacturer’s instructions with minor modifications described previously^9,10^. Muscle non-heme iron: Gastrocnemius samples (≈30 mg) were snap-frozen, weighed, and homogenized on ice in 3 volumes of acid lysis buffer (provided) containing 0.5 % Triton X-100 and 1× protease inhibitor. After centrifugation (12,000 g, 4 °C, 10 min), 100 µL supernatant was mixed with 100 µL iron releasing agent (supplied) and incubated at 95 °C for 30 min to liberate protein-bound iron. After cooling, 50 µL of the mixture was transferred to a 96-well plate, followed by 50 µL chromogen reagent (ferrozine-based). Absorbance at 562 nm was recorded within 5 min using a microplate reader (BioTek Synergy H1). Iron concentration was calculated against a Fe²⁺ standard curve (0-1.5 µg mL⁻¹) prepared in parallel and normalized to tissue wet weight (µg Fe g⁻¹ tissue). Serum iron: Blood was collected via cardiac puncture, allowed to clot at RT for 30 min, and centrifuged (2,000 g, 4 °C, 15 min) to obtain serum. 50 µL serum was mixed with 50 µL iron releasing agent, heated at 95 °C for 30 min, cooled, and processed identically to tissue samples. Results are expressed as µg Fe dL⁻¹ serum.All samples were run in duplicate; intra-assay CV < 5 %. Blank and standard curves were included in each run to ensure linearity (R² ≥ 0.998).

11. **ATP assay**

ATP levels were measured using the Beyotime ATP Assay Kit (S0026) following the manufacturer's instructions with minor modifications^11^. Muscle tissue: Gastrocnemius (~20 mg) was rapidly dissected, snap-frozen, and homogenized in 200 µL ice-cold ATP lysis buffer (provided) with a bead-mill (4 °C, 60 s). After centrifugation (12,000 g, 4 °C, 5 min), 20 µL supernatant was mixed with 100 µL ATP working solution (freshly prepared by diluting luciferase reagent 1:9). Relative light units (RLU) were read immediately (2-s delay, 10-s integration) on a Synergy H1 microplate reader. ATP concentration (nmol mg⁻¹ wet weight) was calculated from an ATP standard curve (0.01-10 µM) run in parallel and normalized to tissue weight. MuSCs: Cells (5 × 10⁴ per well, 96-well) were washed with PBS, lysed in 50 µL ATP lysis buffer on ice for 5 min, scraped, and centrifuged (12,000 g, 4 °C, 5 min). Twenty µL supernatant was assayed as above. Results were expressed as nmol ATP mg⁻¹ protein, quantified by BCA assay on the same lysate. All samples were processed within 30 min of lysis to prevent ATP degradation.

12. **Quantitative Real-Time PCR (RT-qPCR)**

Total RNA from FACS-sorted MuSCs (CD45^-^CD11b^-^CD31^-^Sca1^-^Vcam^+^, 5×10^5^-1×10^6^ cells) or treated MuSCs (1×10^6^-5×10^6^ cells) was extracted using TRIzol Reagent (15596026, Invitrogen, USA). RNA was precipitated with glycogen carrier, washed twice with 75 % ethanol, and dissolved in 20 µL RNase-free H₂O. Reverse transcription was performed with the Takara cDNA synthesis kit (RR047Q, Takara, Japan) in a 20 µL reaction. Real-time PCR was carried out with TB Green® Premix Ex Taq™ II (RR820A, Takara, Japan) on a StepOnePlus Real-Time PCR System (40 cycles: 95 °C 15 s, 60 °C 30 s). Gapdh served as the internal control and gene expression was quantified using the 2^−ΔΔCt^ method. Primers were used as follow:

| Primers | Forward (5’-3’) | Reverse (5’-3’) |
| --- | --- | --- |
| Bnip3 | CGCACAGCTACTCTCAGCAT | GTGGAAGTTGTCAGACGCCT |
| Atg5 | ATCCAAGGATGCGGTTGAGG | CGGTTGATGGCCCAAAACTG |
| Pink1 | TATCTCGGCAGGTTCCTCCA | GATGTTAGGGTGTGGGGCAA |
| Park2 | CCTGCAAACAAGCAACCCTC | CCTGTTGCTGGTGATCAGGT |
| Gpx4 | ATAAGAACGGCTGCGTGGTGAAG | TAGAGATAGCACGGCAGGTCCTTC |
| Slc7a11 | CTATTTTACCACCATCAGTGCG | ATCGGGACTGCTAATGAGAATT |
| Facl4 | TGGTCAGGGATATGGGCTGA | CTGGCCACCGATCACAATCT |
| Ncoa4 | GAGGTGTAGTGATGCACGGAG | GACGGCTTATGCAACTGTGAA |
| Cdk4 | GCCTGTGTCTATGGTCTG | AAGCAGGGGATCTTACGC |
| Ki67 | TGCTTGCTGGAGTTCTGACTGATTC | AGCCTGTGGAAGACCTGACTGG |
| Pcna | TCATCAATGAGGCCTGCTGG | GGACATGCTGGTGAGGTTCA |
| Ccna2 | AAGAGAATGTCAACCCCGAAA | ACCCGTCGAGTCTTGAGCTT |
| Gapdh | ACGGCAAGTTCAACGGCACAG | GAAGACGCCAGTAGACTCCACGAC |

13. **Western Blotting (WB)**

Mouse skeletal-muscle samples or treated MuSCs were lysed in ice-cold RIPA buffer containing PMSF protease inhibitor (Beyotime, ST506) and phosphatase inhibitor (Beyotime, P1045). Proteins were separated on 10 % or 12.5 % (w/v) SDS-PAGE gels and transferred to PVDF membranes (Bio-Rad, USA). After blocking with 5 % non-fat milk, the membranes were incubated overnight at 4 °C with primary antibodies. Following three TBST washes, the membranes were incubated with appropriate HRP-conjugated secondary antibodies for 1 h at room temperature. Blots were visualized using a chemiluminescence kit (MedChemExpress, HY-K2005) and quantified with ImageJ. Primary and secondary antibodies used are listed below: Anti-Gapdh (1:10000, Proteintech, HRP-60004), anti-β-actin (1:100000, Proteintech, 66009-1-Ig), anti-Ampk (1:800, Zenbio, R23314), anti-Phospho-Ampk- Thr183/Thr172 (1:1000, Zenbio, 340763), anti-Tsc2 (1:4000, Proteintech, 24601-1-AP; 1:1000, Selleck, F0311), anti-Phospho-Tsc2-Ser1387 (1:8000, Proteintech, 80698-1-RR; 1:1000, Selleck, F1948), anti-mTor (1:1000, Zenbio, 380411), anti-Phospho-mTo-Ser2448 (1:2000, Zenbio, R25033), anti-Pink1 (1:800, Santa Cruz Biotechnology, sc-517353), anti-Parkin (1:800, Santa Cruz Biotechnology, sc-32282), anti-Lc3b (1:800, Proteintech, 18725-1-AP) and anti-Gpx4 (1:2000, Zenbio, R381958), anti-Slc7a11 (1:1000, Zenbio, R382036), Anti-Ncoa4 (1:2000, Zenbio, 680442), anti-Fth1 (1:2000, Zenbio, R381204), and anti-Tfr1 (1:2000, Zenbio, R381603); Anti-Rabbit IgG (H+L) HRP (1:100000, MultiSciences, GAR007); Anti-Mouse IgG (H+L) HRP (1:100000, MultiSciences, GAM007).

14. **Hindlimb grip-strength test**

Hindlimb grip strength was evaluated with a digital grip-strength meter (Shanghai Xinruan Technology, China) as previously described^12^. Briefly, mice were allowed to grasp the metal grid (3 mm diameter bars, 5 mm spacing) with the hind paws only while being gently pulled away by the tail in a horizontal plane. The peak force (g) recorded at the moment of release was registered automatically by the sensor. Each animal was tested 5 consecutive times with a 30-s rest between trials; the average value was taken as the individual hind-limb grip-strength peak. All measurements were performed between 09:00 and 11:00 by an investigator blinded to treatment, and the apparatus was cleaned with 70 % ethanol between subjects to remove scent cues.

15. **Rotarod test**

Motor coordination was assessed using the rotarod paradigm according to published protocols^13^. During training, animals were placed on the rotating rod at 4 rpm for 10 min per session on three consecutive days. For testing, the rod accelerated from 4 rpm at 0.1 rpm s⁻¹, and latency to fall was recorded. Three trials per animal were conducted with 10-min inter-trial breaks.

16. **Exhaustive Swim test**

As described previously, a weight-loaded swimming test was used to evaluate exercise capacity^14,15^. Briefly, Individual animals were weighed and a lead pellet equal to 15% body weight was fixed at the tail base. Mice swam singly in a 50×50×50 cm tank maintained at 40 cm depth and 25 ± 1°C. Pre-test adaptation consisted of three consecutive days of 3-min swimming sessions. During the trial, exhaustion was defined as failure to surface within 7s; the animal was then promptly retrieved and towel-dried. Gentle agitation with a glass rod was applied to keep the mouse swimming continuously until exhaustion, and total swimming time was recorded.

**References:**

1. Cusmano AA, VanGenderen CA, Lorenz TO, Yang Y, Chang NC. Efficient Isolation and Ex Vivo Differentiation of Murine Satellite Cells from Healthy and Dystrophic Muscle. In: Xenotransplantation. Springer US: New York, NY; 2025. pp. 37–54.

2. Barajaa MA, Otsuka T, Laurencin CT. A novel protocol for the direct isolation of a highly pure and regenerative population of satellite stem cells. Proc Natl Acad Sci USA 2025;122.

3. Musarò A, Carosio S. Isolation and Culture of Satellite Cells from Mouse Skeletal Muscle. In: Xenotransplantation. Springer New York: New York, NY; 2017. pp. 155–167.

4.	Luo A, Wang R, Gong J, Wang S, Yun C, Chen Z et al. Syntaxin 17 Translocation Mediated Mitophagy Switching Drives Hyperglycemia‐Induced Vascular Injury. Adv Sci 2025;12.

5. Chen S, Ding H, Yao X, Xie L. Isolation and Culture of Single Myofiber and Immunostaining of Satellite Cells from Adult C57BL/6J Mice. Bio Protoc 2019;9.

6. Xie L, Yin A, Nichenko AS, Beedle AM, Call JA, Yin H. Transient HIF2A inhibition promotes satellite cell proliferation and muscle regeneration. J Clin Invest 2018;128:2339–2355.

7.	Liu L, Cheung TH, Charville GW, Rando TA. Isolation of skeletal muscle stem cells by fluorescence-activated cell sorting. Nat Protoc 2015;10:1612–1624.

8. Lin H, Yin L, Liu W, Li R, Jiang T, Yang M et al. Muscle‐Derived Small Extracellular Vesicles Mediate Exercise‐Induced Cognitive Protection in Chronic Cerebral Hypoperfusion. Adv Sci 2025; e2410209.

9. Wu Y, Huang T, Li X, Shen C, Ren H, Wang H et al. Retinol dehydrogenase 10 reduction mediated retinol metabolism disorder promotes diabetic cardiomyopathy in male mice. Nat Commun 2023;14.

10. Pei Z, Qin Y, Fu X, Yang F, Huo F, Liang X et al. Inhibition of ferroptosis and iron accumulation alleviates pulmonary fibrosis in a bleomycin model. Redox Biol 2022;57:102509–102509.

11. Wang X, Wei Z, Gu M, Zhu L, Hai C, Di A et al. Loss of Myostatin Alters Mitochondrial Oxidative Phosphorylation, TCA Cycle Activity, and ATP Production in Skeletal Muscle. IJMS 2022;23:15707.

12. Deng Z, Song C, Chen L, Zhang R, Yang L, Zhang P et al. Inhibition of CILP2 Improves Glucose Metabolism and Mitochondrial Dysfunction in Sarcopenia via the Wnt Signalling Pathway. J Cachexia Sarcopenia Muscle 2024 doi:10.1002/jcsm.13597.

13. Fang X, Ding H, Chen Y, Wang Q, Yuan X, Zhang C et al. Wireless Optogenetic Targeting Nociceptors Helps Host Cells Win the Competitive Colonization in Implant‐Associated Infections. Small Methods 2024;8:e2400216.

14. Huang W-C, Chiu W-C, Chuang H-L, Tang D-W, Lee Z-M, Wei L et al. Effect of Curcumin Supplementation on Physiological Fatigue and Physical Performance in Mice. Nutrients 2015;7:905–921.

15. Wu R-E, Huang W-C, Liao C-C, Chang Y-K, Kan N-W, Huang C-C. Resveratrol Protects against Physical Fatigue and Improves Exercise Performance in Mice. Molecules 2013;18:4689–4702.

Supplementary Figures

**Figure S1. Denervated skeletal muscle exhibits Ca²⁺ dysregulation, enhanced autophagy, and ferroptosis**

A: The volcano plot illustrates DEGs in skeletal muscle from DN and Sham, calcium-metabolism genes are highlighted in blue, autophagy genes in red.

B-D: GO enrichment analysis of biological process (GO_BP, B), molecular function (GO_MF, C) and cellular component (GO_CC, D) for up-regulated DEGs and down-regulated DEGs. Dot size indicates gene count; color intensity indicates -log10(*P*-value).

E: Quantification of mean Ca²⁺ fluorescence intensity in isolated gastrocnemius (Gas) fibers of Sham versus DN mice, n=5 mice per group.

F: As assessed in Figure 1D, the abundance of damaged mitochondria in Gas TEM sections was quantified and compared between Sham and DN groups, n=5 mice per group.

G: Quantitative comparison of serum iron levels and Gas non-heme iron content between Sham and DN mice. n=5 mice per group.

H: As assessed in Figure 1E, quantification of Fe^2+^ fluorescence in isolated Gas fibers from DN and Sham mice, n=5 mice per group.

I: As determined from Figure 1F, the Oil Red O-positive area in Gas cryosections was calculated and compared between Sham and DN mice, n=5 mice per group.

J: RT-qPCR analysis of ferroptosis-related gene expression (Gpx4, Slc7a11, Facl4, Ncoa4) in Gas muscle from Sham and DN mice, n=4 mice per group.

Statistical analysis: Two-sided unpaired t-test. Data are presented as mean ± SEM.

**Figure S2. Denervation impairs muscle satellite cell (MuSC) proliferation by 2 weeks**

A: The gating strategy for flow cytometric analysis or sorting of MuSCs from mouse skeletal muscle, L: low expression, H: high expression.

B & C: According to Figure 2D and 2E, statistical analysis of the proportion of MuSCs in gastrocnemius (Gas) between Sham and denervated (DN) mice at 2 weeks (B) or 4 weeks (C) post-surgery, n=5 mice per group.

D: As assessed in Figure 2F, the number of Pax7^+^ MuSCs in Gas muscle was calculated between Sham and DN mice, n=5 mice per group.

E: The changes in the number of MuSCs in the Gas of mice at 2, 4, 6, and 8 weeks after denervation, presented as a proportion of total cells, n=5 mice per group.

F: Statistical analysis of the difference in wet weight of Gas muscle between Sham and DN groups, n=5 mice per group.

G: Representative immunofluorescence images of neuromuscular junctions in Gas muscle of Sham and DN mice, scale bar, 100 μm.

H: Representative immunofluorescence micrographs and statistical analysis of type I and II fiber in Gas muscle, comparing Sham versus DN mice, n=5 mice per group.

I: Representative hematoxylin and eosin (H&E) staining images of gastrocnemius (Gas) muscle sections from Sham and DN mice, with quantitative analyses of myofiber density, mean myofiber cross-sectional area (CSA), and myofiber diameter distribution. Scale bar, 100 μm.

Statistical analysis: (A–D, F, H, and myofiber density and mean CSA in I) two-sided unpaired Student’s t-test; (myofiber diameter distribution in I) two-way ANOVA. Data are presented as mean ± SEM.

**Figure S3. Ca^2+^ overload induces Mitophagy in MuSCs via the Ampk-Tsc2-mTorc1 axis**

A: According to Figure B, statistical analysis of the expression of Ampk, p-Ampk, Tsc2, p-Tsc2, mTorc1, and p-mTorc1 in Gas of Sham and DN mice, n=3 biological replicates.

B: According to Figure D, statistical analysis of the difference in mean Ca^2+^ fluorescence intensity among MuSCs in Ctrl, A23187, and Bapta-am groups, n=4.

C: According to Figure E, quantitative analysis of the green/red fluorescence intensity ratio (%) in MuSCs from Ctrl, A23187, and Bapta_am groups, n=5.

D: Difference in ATP production level of MuSCs among Ctrl, A23187, and Bapta_am groups, n=4.

E: RT-qPCR quantifies the expression differences of mitophagy genes (Bnip3, Atg5, Pink1, Park2) in MuSCs among Ctrl, A23187, and Bapta_am groups, n=4.

Statistical analysis: One-way analysis of variance test, ANOVA. Data are presented as mean ± SEM.

**Figure S4. Excessive mitophagy aggravates Fe^2+^ accumulation and triggers ferroptosis**

A: According to Figure 4A, quantitative analysis of Fe^2+^-metabolism-related proteins (Fth1 and Tfr1) in Gas muscle of Sham and DN groups, n=3.

B: Representative DCFH-DA staining for reactive oxygen species (ROS) in isolated Gas myofibers and statistical analysis of mean ROS fluorescence intensity in single myofibers of Sham and DN groups, scale bar,100μm, n=5.

C: According to Figure 4E, statistical analysis of the differences in the proportion of ROS⁺ MuSCs among Ctrl, A23187, and Mdivi-1 groups.

D: Representative Fe^2+^ staining and statistical analysis of mean fluorescence intensity in MuSCs treated with vehicle (Ctrl group), 5 μM A23187 (A23187 group), or A23187 plus 5 μM Mdivi-1 (Mdivi-1 group). Scale bar, 50 μm, n=5.

E: According to Figure 4F, quantitative analysis of green/red fluorescence intensity (%) in MuSCs from Ctrl, A23187 and Mdivi-1 groups, red fluorescence represents the cellular aggregate LPO and green fluorescence indicates the monomeric LPO, n=5.

F: Immunoblots and statistical analysis of the expression of ferroptosis-related proteins (Gpx4, Slc7a11, and Ncoa4) in MuSCs among Ctrl, A23187 and Mdivi-1 groups, n=3.

G: RT-qPCR quantifies the expression of Pink1 in MuSCs treated with scrambled control siRNA (Scr) or Pink1-targeting siRNA (siPink1), n=5.

H: Immunoblots and statistical analysis of the expression of Fe^2+^-metabolism-related proteins (Fth1 and Tfr1) in MuSCs among Scr_Ctrl, Scr_A23187, siPink1_Ctrl, and siPink1_A23187 groups.

I: Representative Fe^2+^ staining and statistical analysis of mean Fe²⁺ fluorescence intensity in MuSCs among Scr_Ctrl, Scr_A23187, siPink1_Ctrl, and siPink1_A23187 groups, scale bar, 50 μm, n=5.

J: Immunoblots and statistical analysis of the expression of ferroptosis markers (Gpx4, Slc7a11, and Ncoa4) in MuSCs among Scr_Ctrl, Scr_A23187, siPink1_Ctrl, and siPink1_A23187 groups, n=3.

Statistical analysis: (A, B and G) Two-sided unpaired t-test, (C-F, and H-J) One-way analysis of variance test, ANOVA. Data are presented as mean ± SEM.

**Figure S5. Experimental design for the application of mitochondrial transplantation therapy (MTT) in vivo and in vitro.**

A: Schematic diagram of mitochondrial isolation, purification and transplantation.

B: Representative micrographs of MitoTracker-stained mitochondria, scale bar, 100 μm.

C: Representative fluorescence image of in-vivo mitochondrial transplantation; red: MitoTracker deep red FM, green: α-Tubulin, scale bar, 100 μm.

D: Representative image of mitochondrial uptake after ex-vivo mitochondrial transplantation; red: MitoTracker deep red FM, scale bar, 100 μm.

**Figure S6. Mitochondrial transplantation mitigates Ca²⁺ overload-induced mitophagy to suppress ferroptosis in MuSCs**

A: According to Figure 5A, comparison of mean Ca²⁺ fluorescence intensity in MuSCs among Ctrl, A23187 and Mito groups, n=4.

B: According to Figure 5B, statistical analysis of JC-1 in MuSCs from Ctrl, A23187 and Mito groups, expressed as green/red fluorescence intensity (%), n=6.

C: Differences in ATP production of MuSCs among the Ctrl, A23187, and Mito groups, n=5.

D: According to Figure 5E, statistical analysis of mean fluorescence differences of Fe^2+^ in MuSCs among the Ctrl, A23187 and Mito groups, n=5.

E: Representative ROS fluorescence staining and statistical analysis of mean fluorescence intensity of ROS in Ctrl, A23187 and Mito groups MuSCs, scale bar, 100 μm, n=5.

F: Representative flow-cytometric histograms and statistical analysis of the differences in the proportion of ROS^+^ MuSCs in the Ctrl, A23187 and Mito groups, n=5.

G: Representative LPO fluorescence staining and statistical analysis of green/red fluorescence intensity (%) in MuSCs from Ctrl, A23187 and Mito groups, red fluorescence indicates non-oxidized lipids, green fluorescence indicates lipid hydroperoxides, scale bar, 100 μm, n=4.

Statistical analysis: One-way analysis of variance test, ANOVA. Mean ± SEM.

**Figure S7. Flow cytometric analysis of mitophagy flux using mt-Keima reporter after denervation injury.**

A: Representative gating strategy for flow cytometric quantification of mitophagy flux using mt-Keima fluorescence. Cells were sequentially gated based on forward and side scatter (FSC/SSC) to identify target cells, followed by FSC-height/FSC-area analysis to exclude doublets and select single cells. Mitophagy flux was determined by analyzing mt-Keima fluorescence signals in the PE and FITC channels, representing mitochondria exposed to acidic lysosomal environments and mitochondria maintained in non-acidic compartments, respectively.

B: Representative mt-Keima flow cytometry plots and quantitative analysis of mitochondrial autophagy flux at 2 weeks after denervation. The PE-positive mitochondrial population represents mitochondria localized within acidic autolysosomal compartments and indicates active mitophagic flux. The FITC mean fluorescence intensity of mito-mKeima-labeled mitochondria was analyzed to estimate the preserved mitochondrial pool. n=5 mice per group.

Statistical analysis: One-way analysis of variance test, ANOVA. Data are presented as mean ± SEM.

**Figure S8. MTT attenuates denervation-induced ferroptotic responses and restores muscle regenerative activity.**

A: Representative ROS fluorescence staining and statistical analysis of ROS fluorescence intensity in single myofibers of Gas among S_PBS, DN_PBS and MTT groups, scale bar, 40μm, n=5 mice per group.

B: Statistical analysis of serum iron levels and Gas non-heme iron content between the S_PBS, DN_PBS and MTT groups, n=5 mice per group.

C-F: RT-qPCR quantified the expression of ferroptosis-related genes (Gpx4, Slc7a11, Facl4 and Ncoa4) in Gas of the S_PBS, DN_PBS, and MTT mice, n=5 mice per group.

G-J: RT-qPCR quantified the expression of cell proliferation-related genes (Cdk4, ki67, Pcna, Ccna2) in Gas of the S_PBS, DN_PBS and MTT mice, n=5 mice per group.

Statistical analysis: One-way analysis of variance test, ANOVA. Data are presented as mean ± SEM.
